# Immediate Growth Control in Response to Boiling Histotripsy is Prognostic for Intratumoral Immune Activation

**DOI:** 10.1002/advs.76722

**Published:** 2026-07-24

**Authors:** Lydia E. Kitelinger, Matthew R. DeWitt, Carly M. Van Wagoner, Claire A. Conarroe, Charles C. Funk, Aaron B. Streit, AeRyon Kim, Richard J. Price, Timothy N. J. Bullock

**Affiliations:** ^1^ Department of Pathology University of Virginia Charlottesville Virginia USA; ^2^ Department of Radiology & Medical Imaging, Department of Biomedical Engineering University of Virginia Charlottesville Virginia USA; ^3^ Department of Microbiology, Immunology, and Cancer Biology University of Virginia Charlottesville Virginia USA

**Keywords:** antigen‐presenting cells, boiling histotripsy, cavitation, melanoma, tumor antigen, tumor microenvironment

## Abstract

Despite the clear efficacy of immune checkpoint blockade, a subset of melanoma patients receives no clinical benefit from treatment. Boiling histotripsy (BH), a mechanical form of focused ultrasound, allows for non‐invasive and non‐ionizing tissue destruction. As mechanically fractionating tumors with BH may potentiate T cell‐mediated anti‐tumor responses, we addressed gaps in knowledge regarding the impact BH has on antigen‐presenting cell (APC) presence in the tumor microenvironment (TME), antigen acquisition, and conventional dendritic cell (cDC) activation states in settings where BH does or does not induce a primary tumor growth response. When BH treatment immediately inhibited melanoma outgrowth, APC presence in the TME was significantly reduced, tumor antigen acquisition amongst APCs remained elevated when compared to controls, and intratumoral immune activation was observed distinctly in the cDC1 subset. Cavitation scoring based on real‐time B‐mode imaging signatures characteristic of BH‐induced tissue destruction demonstrated that BH‐mediated destruction strongly correlates with tumor antigen presence in the draining lymph nodes 24 h post‐ablation. Collectively, these findings reveal that immediate BH‐driven tumor growth control in a melanoma model stratifies distinct intratumoral immune states; positioning early growth response as a biologically informative prognostic indicator of BH‐induced intratumoral immune activation.

## Graphical abstract

Boiling Histrotripsy (BH), a mechanical form of focused ultrasound (FUS), allows for non‐invasive and non‐ionizing tissue destruction. Utilizing a BH treatment protocol that yielded bifurcating primary tumor growth responses, we found that immediate BH‐induced tumor growth control identifies tumors with acute reductions in antigen‐presenting cells (APC), increased antigen acquisition by APCs, cDC1 activation, and enhanced tumor antigen dissemination to the tumor‐draining lymph nodes (TDLN). This suggests early growth response is a prognostic indicator of BH‐mediated immune activation.

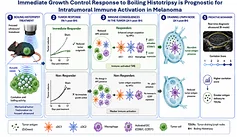

## 1 Introduction

Melanoma remains the deadliest type of skin cancer. While the incidence rate for melanoma continues to rise, there has been success over the last decade in reducing the mortality rate [1, 2]. Much of this success can be attributed to the advent of both targeted therapies and immune checkpoint blockade (ICB) [3]. Despite this promise, there remains a large subset of patients, 40%–60%, that do not respond to ICB [4, 5, 6], suggesting that the clinical deployment of these therapeutics alone is insufficient to provide benefit to all patients. This therapeutic gap is likely due to the lack of immune infiltration in these tumors, and highlights the need for complementary strategies that not only reduce tumor burden but also promote productive anti‐tumor immunity with combinatory ICB [7]. Accordingly, there is increasing interest in locoregional therapies, such as ablation, that not only are employed to reduce primary tumor burden, but also reshape the tumor microenvironment (TME) in ways that may promote anti‐tumor immune responses and improve sensitivity and responsiveness to ICB.

Boiling histotripsy (BH) is one such ablation modality. BH is a mechanical form of focused ultrasound (FUS), a non‐invasive and non‐ionizing energy source that concentrates acoustic waves into a small therapeutic volume. By deploying high‐pressure MHz ultrasound pulses on the order of magnitude of milliseconds focused into a tumor, tissue fractionization is induced through endogenous bubble formation (boiling) and cavitation that undergo oscillation and ultimately implosion [8]. BH is an attractive therapeutic modality not only due to the non‐invasive nature of FUS, but also because this is a non‐thermal based approach. The non‐thermal mechanism of tissue destruction is believed to achieve sharp treatment margins between the ablated and non‐ablated tissue, as there is no dispersal or residual heating away from the treated region [9]. Such precision is important when treating cancerous lesions that are in close proximity to central nerves or major organs and blood vessels that may be damaged by thermal stress. Pre‐clinical studies have demonstrated that treating solid tumors with mechanically ablative FUS, such as BH, can result in constrained primary and distal tumor growth – deemed an abscopal response [10, 11, 12]. While BH is a promising debulking and immune‐stimulating treatment, it has not been shown to be curative in pre‐clinical melanoma models, and the immunologic consequences of variable tumor growth responses remain poorly defined [10, 12]. Therefore, it is crucial to understand how BH is impacting the immunogenicity of melanomas as a monotherapy so that we can explore efficacious combinatorial regimens that integrate with BH to combat this disease, while limiting toxicity that can arise when treating patients with ICB and immune agonists [3].

In previous studies, we assessed the immunological effects of BH by evaluating tumor antigen acquisition by antigen‐presenting cells (APCs) in the tumor‐draining lymph nodes (TDLNs), the site of initial T‐cell priming, in a murine melanoma cell line (B16F10) stably transduced to express the model fluorescent antigen from *Zoanthus sp*. – ZsGreen (ZsG) for antigen tracking [10]. While we examined the presence of ZsG antigen in many APCs post BH, we focused specifically on conventional dendritic cells (cDCs) as they are the professional APCs capable of eliciting robust adaptive T‐cell responses [13]. cDCs are comprised of functionally specialized subsets, and can be classified into cDC1s and cDC2s. cDC1s can directly cross‐present antigen to CD8^+^ T‐cells, while cDC2s specialize in the presentation of antigen to CD4^+^ T‐cells, but they have also been shown to cross‐present antigen to CD8^+^ T‐cells under certain conditions [14].

Despite these previous findings, there remains a significant knowledge gap pertaining to the effects of BH on APC presence and acquisition of tumor‐derived antigen in the TME itself shortly after treatment, or the temporal kinetics of any such effects. Acquiring such knowledge is crucial for the field to improve upon the therapeutic potential of non‐thermal ablations. The presence of APC subsets in melanoma tumors has prognostic implications. For instance, dendritic cell presence is associated with longer patient survival [15, 16], while macrophage infiltration in early‐stage melanoma is linked to reduced survival time [17]. Additionally, it has been suggested that to stimulate a robust CD8^+^ T‐cell adaptive immune response against a tumor, cDCs in the TME are required to provide a secondary co‐stimulation signal to the T‐cells [18]. Therefore, understanding how BH impacts these APCs will provide insight into whether APC activation and/or their phagocytic potential following BH are rate limiting steps of the Cancer Immunity Cycle [19] and could hinder a robust antitumoral immune response. While others have assessed immune cell presence in tumors at various timepoints following mechanical ablation [11, 12, 20, 21], to date, little is known about tumor antigen liberation and acquisition by APCs in the TME after BH. Thus, we sought to determine whether antigen is being fully sequestered by immunosuppressive phagocytic cells in the TME post BH, or if cDCs can also acquire tumor antigen and upregulate immunostimulatory molecules following BH ablation.

Here, we examined the immunological consequences of a specific BH treatment protocol for a murine melanoma model that yielded a bifurcating primary tumor response through multiple independent studies, with some tumors exhibiting immediate growth control and others showing no control. We found that the efficacy and destructive nature of BH acutely impacts the immune compartment in the TME. In the tumors where outgrowth was immediately constrained, significant reductions in number were observed in APC subsets, including cDC1s, cDC2s, and macrophages. No differences in APC presence were seen when tumor growth was not controlled by 96 h. Tumor growth response was also associated with increased ZsG antigen acquisition and elevated cDC1 activation intratumorally. In addition, we developed a qualitative metric based on real‐time hyperechoic activity observed during BH treatments, as well as the resulting post‐treatment appearance (hypoechoic) via B‐mode imaging, that allows us to predict the degree of antigen accumulation in the TDLNs acutely post ablation independent of BH‐mediated tumor growth inhibition. Collectively, these findings suggest that the degree of BH‐mediated mechanical disruption directly shapes the early immunologic consequences in the TME, which may be beneficial in promoting adaptive immune‐mediated control of tumors with combination approaches.

## 2 Results

### 2.1 Characterization of a BH Treatment Protocol for Murine Melanoma that Yields a Bifurcating Primary Tumor Growth Control Response

The goal of these studies was to understand (i) whether tumor antigen acquisition was unique to the TDLNs, and (ii) how cDCs and other APCs respond to a single dose of mechanical insult via BH and the resulting bolus of antigen present in the TME following ablation. To begin addressing these unknowns, we examined the effects of BH on B16‐ZsG melanoma tumors using conserved FUS parameters [Operational frequency (f_0_ = 2.5 MHz); peak‐negative pressure (PNP = −21 MPa); pulse length (3 ms); pulse repetition frequency (PRF = 4 Hz); sonication time = 10 s/point; treatment spacing = 1 mm; plane separation = 2 mm] (Figure 1A). While the tumor model remained consistent and the average tumor volumes on the day of BH treatment (day 13 post inoculation) across experiments were comparable (Figure 1B–E; Figure S1), this BH treatment paradigm yielded two response groups (i.e., “immediate responders” and “non‐responders”). Experiments in which the BH‐treated cohort exhibited a statistically reduced growth rate constant (*k*) relative to the matched Sham cohort were classified as “immediate responders,” whereas experiments not meeting this criterion were classified as “non‐responders” (detailed in methods section “Immediate responder versus non‐responder criterion”). Parallel 24 h cohorts were retrospectively assigned based on the classification outcome of the corresponding 96 h cohort from the same experiment.

**FIGURE 1:**
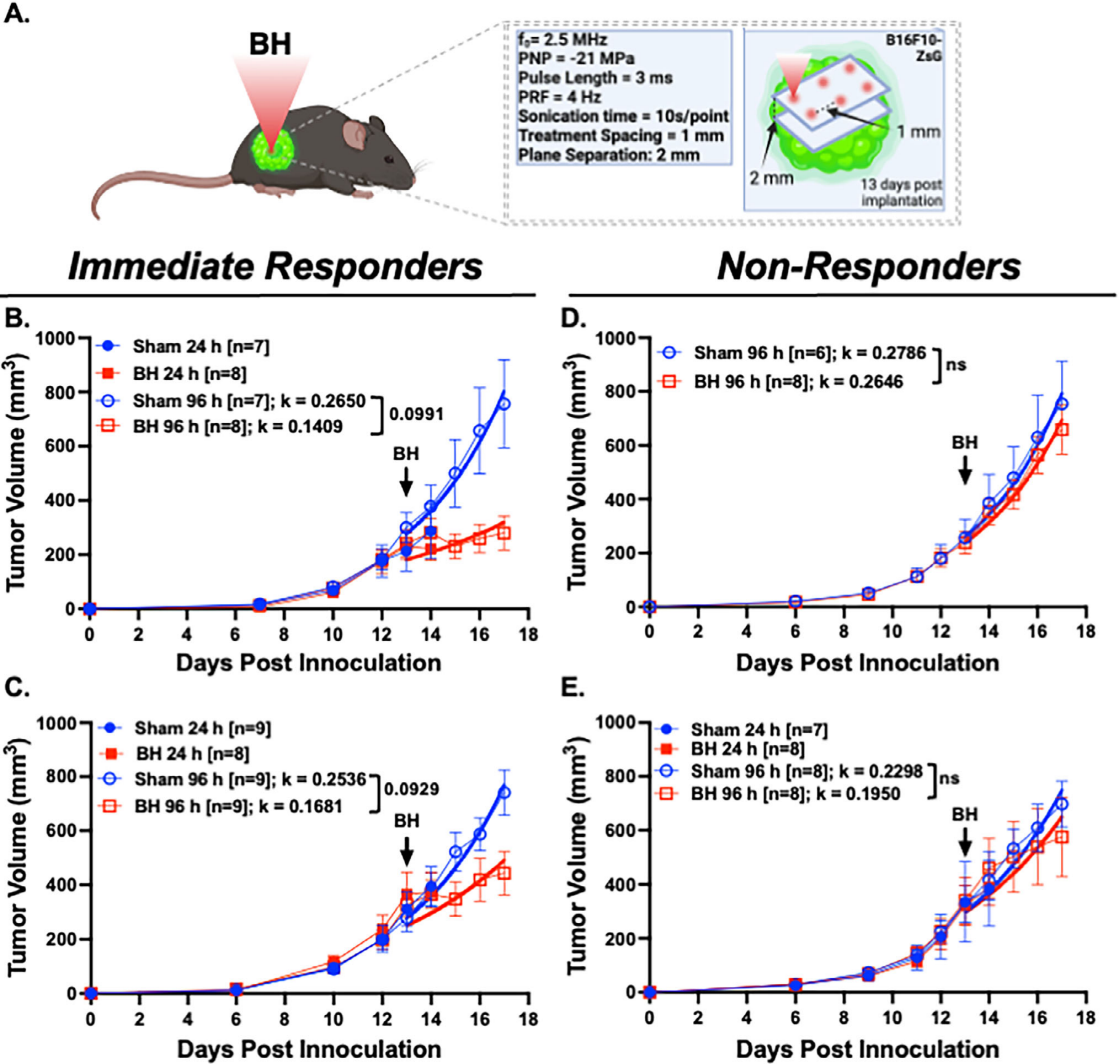
A BH treatment protocol that yields a bifurcating primary tumor growth control response in subcutaneous melanoma. (A) Schematic for conserved boiling histotripsy treatment parameters. (B–E) Average tumor growth curves for B16‐ZsG tumor‐bearing mice treated with Sham or 1mm‐spaced BH on day 13 from four separate experiments. 24 h and 96 h cohorts denote harvest timepoints post BH treatment in which these mice were taken down for subsequent immune analysis. (Nonlinear regression model of exponential growth equation on tumor outgrowth from D13‐D17 for 96 h cohorts. Mixed‐effects model on day 17; ns = nonsignificant) All points represent mean ± SEM.

As tumor outgrowth is only one readout of treatment, we wanted to confirm whether BH ablations – even in the absence of tumor growth control – generated biological perturbations in the immune compartment. To do so, 24 h cohorts from studies where their respective 96 h cohorts either did or did not experience impeded tumor outgrowth via BH were analyzed. Upon harvesting Sham and BH tumors at this timepoint, we observed edema around the BH treated tumors as well as partial liquification of the ablated tumors when compared to the Sham controls, regardless of whether volumetric growth control of the tumors was observed or not (data not shown). Additionally, even in non‐responders, granulocyte presence was significantly increased 24 h post BH ablation – similar to findings observed in the immediate responders (Figure S3; gating strategy provided in Figure S2). This suggests that, although this BH treatment regimen generates both immediate responders and non‐responders, it does consistently induce tissue destruction, as evidenced by the inflammatory response of granulocyte infiltration and edema, as well as physical alterations in tumor consistency visible upon excision.

### 2.2 Immediate Growth Control in Response to BH is Associated with Reduced APC Presence Acutely in the TME

As previously discussed, the presence of immune cell subsets in melanoma tumors can have prognostic implications [17, 22, 23]. Given the differential induction of primary tumor control responses to BH, we next aimed to understand whether there is a relationship between BH‐mediated growth response and APC abundance in the TME. When we examined BH or Sham treated B16‐ZsG tumors 24 h after treatment (Figure 2A–D), immediate responders exhibited a significant reduction in the number and the proportion of cDC1s and cDC2s (Figure 2E,F,I,J; gating strategy provided in Figure S2). We do not believe this reduction is due solely to increased cDC migration to the TDLNs as no significant difference in migratory cDC presence or in their expression of chemokine receptor 7 (CCR7) – the receptor required for cDC trafficking from the TME – was observed in the paired TDLN samples (Figure S5; gating strategy provided in Figure S4) [24, 25]. We also observed a proportional decrease in F4/80^+^ macrophages, however, there was no change in their number (Figure 2M,N); suggesting that the altered proportion is likely due to the influx of granulocytes seen acutely post BH in this setting (Figure S3A–C). Similar effects were observed when we treated another murine melanoma tumor line, YUMM1.7 (Yale University Melanoma Model) [26] – a genomically stable model that expresses the human‐relevant mutations *Braf^V600E^* and loss of *Pten* that was transduced to express ZsG (YUMM‐ZsG) – with BH and induced tumor growth control (Figure S7; gating strategy provided in Figure S6). However, in non‐responders, no reduction or difference in APC presence was observed between Sham and BH cohorts (Figure 2G,H,K,L,O,P). By 96 h post treatment, cDC and macrophages populations had recovered in number and as percentages of the intratumoral immune cells (Figure 3). Similar results were observed in the YUMM‐ZsG tumors (Figure S8). As tumor‐associated macrophages (TAMs) can further be characterized into anti‐tumorigenic (MHCII^+^CD86^+^) and pro‐tumorigenic (MHCII^−^CD86^−^) TAMs, we delineated these subsets from the total F4/80^+^ macrophage population. Overall, we observed minimal changes in macrophage phenotype in the TME 24 and 96 h post BH treatment in both the immediate responder and non‐responder cohorts (Figure S9). While we did find that the MHCII^+^CD86^+^ TAMs were significantly reduced 24 h post BH in the responder tumors (Figure S9A,C), their abundance returned to the Sham baseline by 96 h, mirroring what we observed with other APCs such as the cDC populations (Figures 2 and 3). Taken together, this suggests that APCs are able to quickly repopulate the TME in the growth‐constrained setting following initial reduction.

**FIGURE 2:**
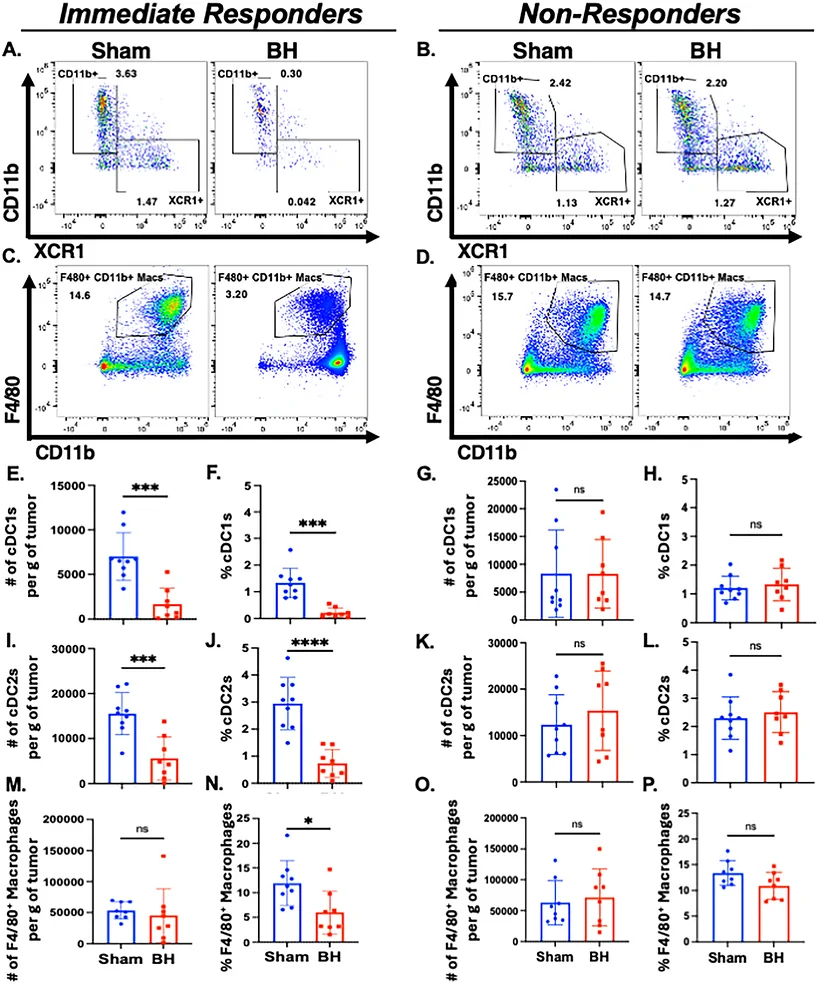
Immediate growth control in response to BH is associated with reduced APC presence acutely in the TME. (A, B) Representative flow plots of cDC1 and cDC2 presence in the TME of immediate responders (A) and non‐responders (B) 24 h post BH. Frequencies shown are of Live/CD45^+^ population. (C, D) Representative flow plots of F4/80^+^ macrophage presence in the TME of immediate responders (C) and non‐responders (D) 24 h post BH. Frequencies shown are of Live/CD45^+^ population. (E, G) Number of cDC1s per g of tumor in immediate responders (E) and non‐responders (G) 24 h post BH. (F, H) Proportion of Live/CD45^+^ cells in the TME that are cDC1s in immediate responders (F) and non‐responders (H) 24 h post BH. (I, K) Number of cDC2s per g of tumor in immediate responders (I) and non‐responders (K) 24 h post BH. (J, L) Proportion of Live/CD45^+^ cells in the TME that are cDC2s in immediate responders (J) and non‐responders (L) 24 h post BH. (M, O) Number of F4/80^+^ macrophages per g of tumor in immediate responders (M) and non‐responders (O) 24 h post BH. (N, P) Proportion of Live/CD45^+^ cells in the TME that are F4/80^+^ macrophages in immediate responders (N) and non‐responders (P) 24 h post BH. (n = 8‐9) Unpaired *t*‐test with Welch's correction: ns = nonsignificant, ∗ *P* < 0.05, ∗∗∗ *P* < 0.001, ∗∗∗∗ *P* < 0.0001; Mean ± SD. ROUT Outliers analysis with Q = 0.1%.

**FIGURE 3:**
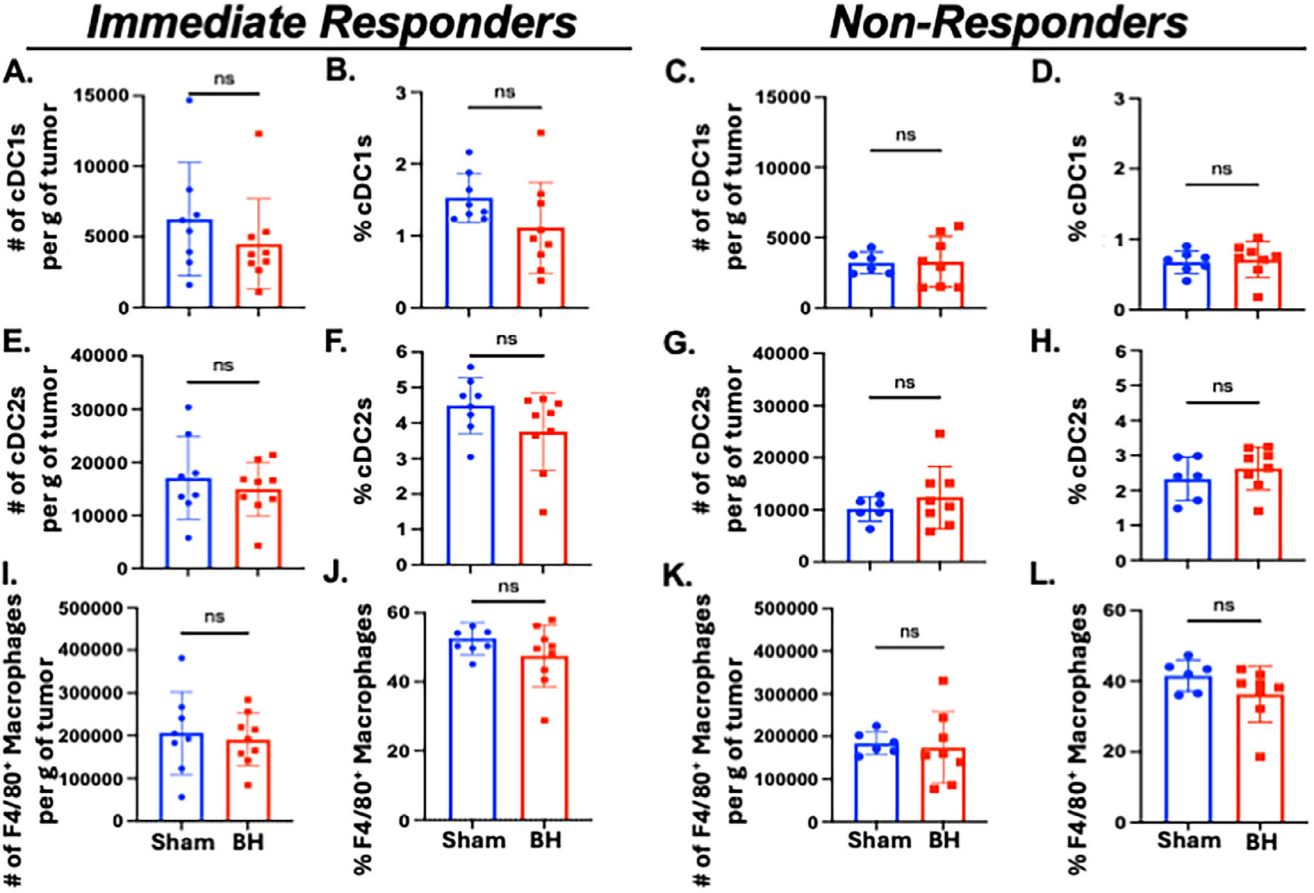
By 96 h post BH, no reduction is observed in APC presence in the TME. (A, C) Number of cDC1s per g of tumor in immediate responders (A) and non‐responders (C). (B, D) Proportion of Live/CD45^+^ cells in the TME that are cDC1s in immediate responders (B) and non‐responders (D). (E, G) Number of cDC2s per g of tumor in immediate responders (E) and non‐responders (G). (F, H) Proportion of Live/CD45^+^ cells in the TME that are cDC2s in immediate responders (F) and non‐responders (H). (I, K) Number of F4/80^+^ macrophages per g of tumor in immediate responders (I) and non‐responders (K). (J, L) Proportion of Live/CD45^+^ cells in the TME that are F4/80^+^ macrophages in immediate responders (J) and non‐responders (L). (n = 7‐8) Unpaired *t*‐test with Welch's correction: ns = nonsignificant; Mean ± SD. ROUT Outliers analysis with Q = 0.1%.

The reduction in immune cell presence in the responder group is not unique to APCs. In both the B16‐ZsG and YUMM‐ZsG models, immediate responder tumors also have a significantly lower abundance of lymphocytes when compared to their respective Sham control cohorts (Figure S10A–E,J–O). No change in lymphocyte populations was evident in non‐responders (Figure S10F–I). Additionally, we observe no change in APC presence in TDLNs and contralateral lymph nodes (cLNs) of immediate responders and non‐responders at this timepoint (Figure S11). While CD8^+^ T‐cell number is also not significantly changed in either setting, the proportion of CD8^+^ T‐cells is reduced in the TDLNs of the immediate responders (Figure S11B), indicating that an immune population not examined is becoming more abundant in the immediate responder TDLNs acutely post BH. Together these data indicate that the efficacy of tumor growth inhibition and the destructive nature of BH have the potential to acutely impact the immune compartment in the melanoma TME.

### 2.3 Increased Tumor Antigen Acquisition is Associated With Immediate Response to BH

While our prior work established that BH ablation results in the dissemination of ZsG tumor antigen to the TDLNs [10], whether BH‐mediated tissue destruction influences antigen acquisition by APCs in the TME following mechanical ablation has not been determined. Given that we have observed a bolus of ZsG antigen in the TDLNs post BH, we predicted that this antigen is also elevated in the TME. Understanding which cells are acquiring this model tumor antigen is important, as the sequestration of antigen by highly phagocytic, pro‐tumorigenic cells, such as TAMs [27], and not cDCs, can decrease the likelihood of initiating a potent antitumorigenic adaptive immune response. To begin addressing antigen uptake, we again utilized the B16‐ZsG model and quantified ZsG acquisition in the TME. This required using a revised gating strategy, as detailed in methods section “ZsGreen analysis in murine melanoma tumors” (Figures S12 and S13). We examined whether BH‐induced tumor growth control was associated with antigen acquisition by APCs in the TME. In immediate responders, BH significantly increased the proportion of cDC1s, cDC2s, and macrophages that contained ZsG tumor antigen 24 h post treatment (Figure 4A–D). No differences in ZsG consumption were observed in APCs in non‐responder tumors when compared to the Sham controls (Figure 4E–H). Interestingly, these significant proportional increases in ZsG capture found in immediate responders persisted and were still observed in the cDC2 and macrophage subsets present 96 h post BH ablation (Figure 5B,C), with a trending increase seen in the cDC1s (Figure 5A). Similar to the 24 h timepoint, there was no enhanced antigen acquisition between the BH and Sham cohorts in non‐responders at 96 h (Figure 5D–F). Analogous results were observed in BH‐treated YUMM‐ZsG tumors (Figure S14). These findings indicate that only in the setting when BH treatment induces immediate primary tumor outgrowth constraint do we observe elevated tumor antigen acquisition by cDCs and macrophages in the TME. Furthermore, these data highlight that tumor antigen released following BH is not entirely sequestered by TAMs, as both the remaining cDC1s and cDC2s experience proportional increases in ZsG acquisition at 24 h. Importantly, when the number of cDCs in the immediate responder tumors recover at 96 h, they contain higher proportions of ZsG tumor antigen than controls; suggesting that they can access residual post‐BH liberated tumor antigen.

**FIGURE 4:**
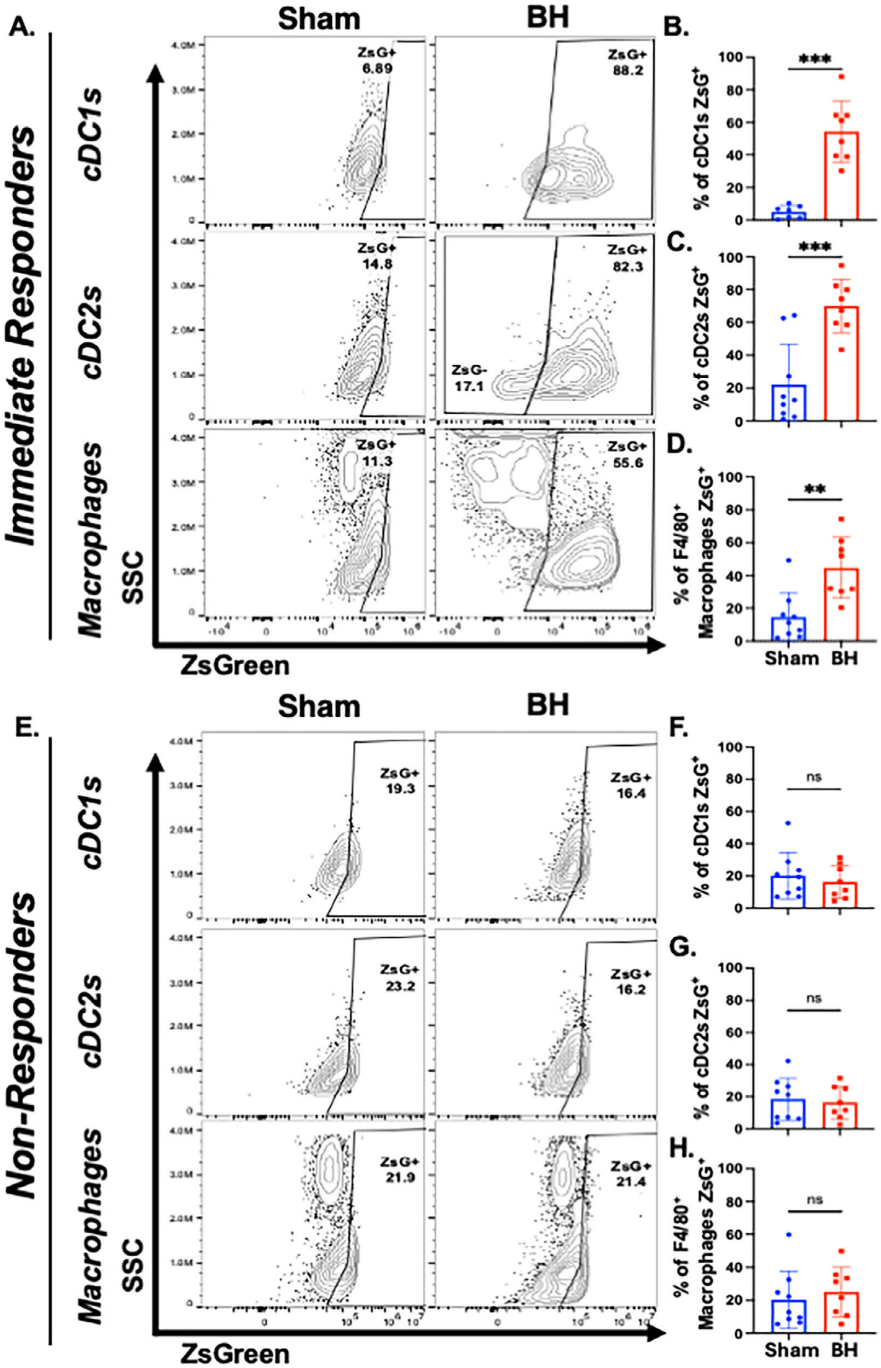
Increased tumor antigen acquisition is associated with immediate response to BH. (A) Representative flow plots from immediate responders 24 h post BH, showing ZsG acquisition in cDC1s (top), cDC2s (middle), and F4/80^+^ macrophages (bottom) in the TME of Sham and BH cohorts. Frequencies shown are of the parent population. (B–D) The proportion of cDC1s (B), cDC2s (C), and F4/80^+^ macrophages (D) that are ZsG^+^ in immediate responder tumors. (E) Representative flow plots from non‐responders 24 h post BH showing ZsG acquisition in cDC1s (top), cDC2s (middle), and F4/80^+^ macrophages (bottom) in the TME of Sham and BH cohorts. Frequencies shown are of the parent population. (F–H) The proportion of cDC1s (F), cDC2s (G), and F4/80^+^ macrophages (H) that are ZsG^+^ in non‐responder tumors. (n = 8‐9) Unpaired t test with Welch's correction: ns = nonsignificant, ∗∗ *P* < 0.01, ∗∗∗ *P* < 0.001; Mean ± SD. ROUT Outliers analysis with Q = 0.1%.

**FIGURE 5:**
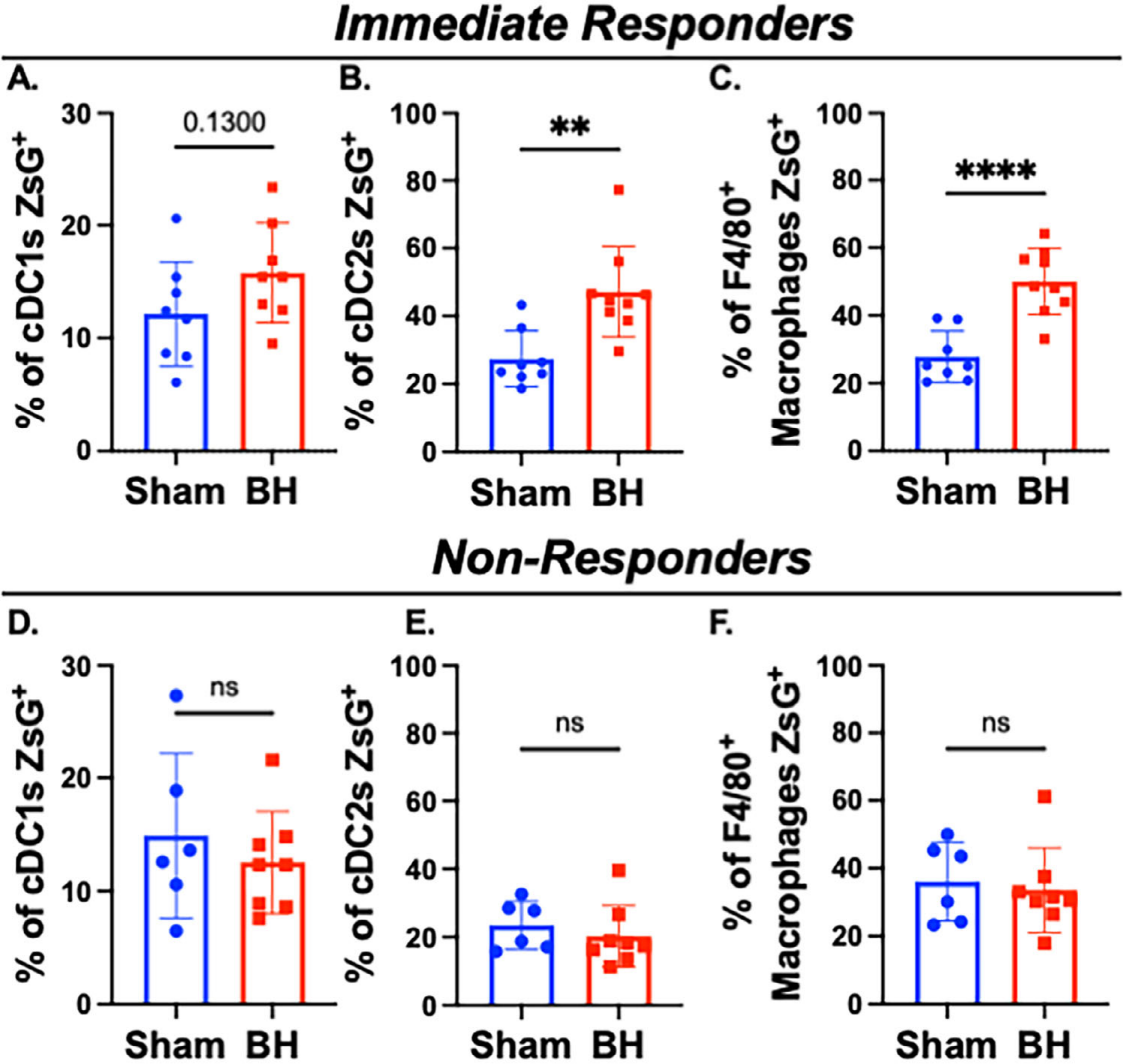
Increased tumor antigen acquisition persists when BH‐induced immediate growth control response is observed. (A–C) Proportion of cDC1s (A), cDC2s (B), and F4/80^+^ macrophages (C) that are ZsG^+^ in immediate responders 96 h post BH. (D–F) Proportion of cDC1s (D), cDC2s (E), and F4/80^+^ macrophages (F) that are ZsG^+^ in non‐responder tumors 96 h post BH. (n = 8‐9) Unpaired t test with Welch's correction: ns = nonsignificant, ∗∗ *P* < 0.01, ∗∗∗∗ *P* < 0.0001; Mean ± SD. ROUT Outliers analysis with Q = 0.1%.

### 2.4 BH Yields Varying Degrees of cDC Phenotypic Activation

We previously reported that BH induces cDC activation in a tumor antigen‐dependent manner in the TDLNs [10]. We specifically focused on cDC activation, as they are the professional APCs capable of eliciting a robust T‐cell response [13]. We have observed the relationship between ZsG acquisition and cDC activation in the TDLNs in both the immediate responder and non‐responder cohorts (Figure S15). This suggests that BH, even if not resulting in immediate tumor growth control, can still induce immunogenic responses capable of enhancing cDC activation. We thus tested whether BH resulted in cDC activation in the TME, and whether tumor growth constraint correlates with the level of cDC activation post BH. We assessed the level of CD86 expression on the cDCs, as this is a co‐stimulatory molecule upregulated on the surface of cDCs as they undergo activation and maturation [28]. We found that, in immediate responders, CD86 was significantly upregulated on the surface of cDC1s in both melanoma models (Figure 6A,B and Figure S17A,B), and a greater proportion of tumoral cDC1s were activated post BH – expressing high levels of CD86 and MHC‐II (Figure 6C and Figure S17C). No differences in activation were observed on the cDC2 population from B16‐ZsG tumors (Figure 6A,D,E), however, cDC2s from immediate responder YUMM‐ZsG tumors did display higher levels of activation 24 h post BH (Figure S17A,D,E). Contrary to immediate responders, CD86 elevation was not seen on the cDC1s for non‐responders (Figure 6F–H and Figure S16A,B). It is of note that, while augmentation of CD86 expression was not observed on cDC1s in either non‐responder experiment, the degree of cDC2 activation varied between experiments. In one, cDC2s experienced increased CD86 intensity and CD86^hi^MHC‐II^hi^ expression 24 h following BH (Figure 6F,I,J), while no significant difference in cDC2 activation was seen in the other (Figure S16C,D). It is possible that the degree of activation induced on cDC2s in the tumors where BH did not control primary melanoma growth is not as potent as what we observe on the cDC1s in the constrained tumors. Additionally, by 96 h post treatment, no differences in cDC activation were found in the BH cohorts from either immediate responders or non‐responders (Figures S16E–L and S17F–I). Altogether, these findings highlight that the varying degrees of BH‐mediated tumor growth constraint are associated with differential acute activation of cDCs – with constrain promoting cDC1 activation.

**FIGURE 6:**
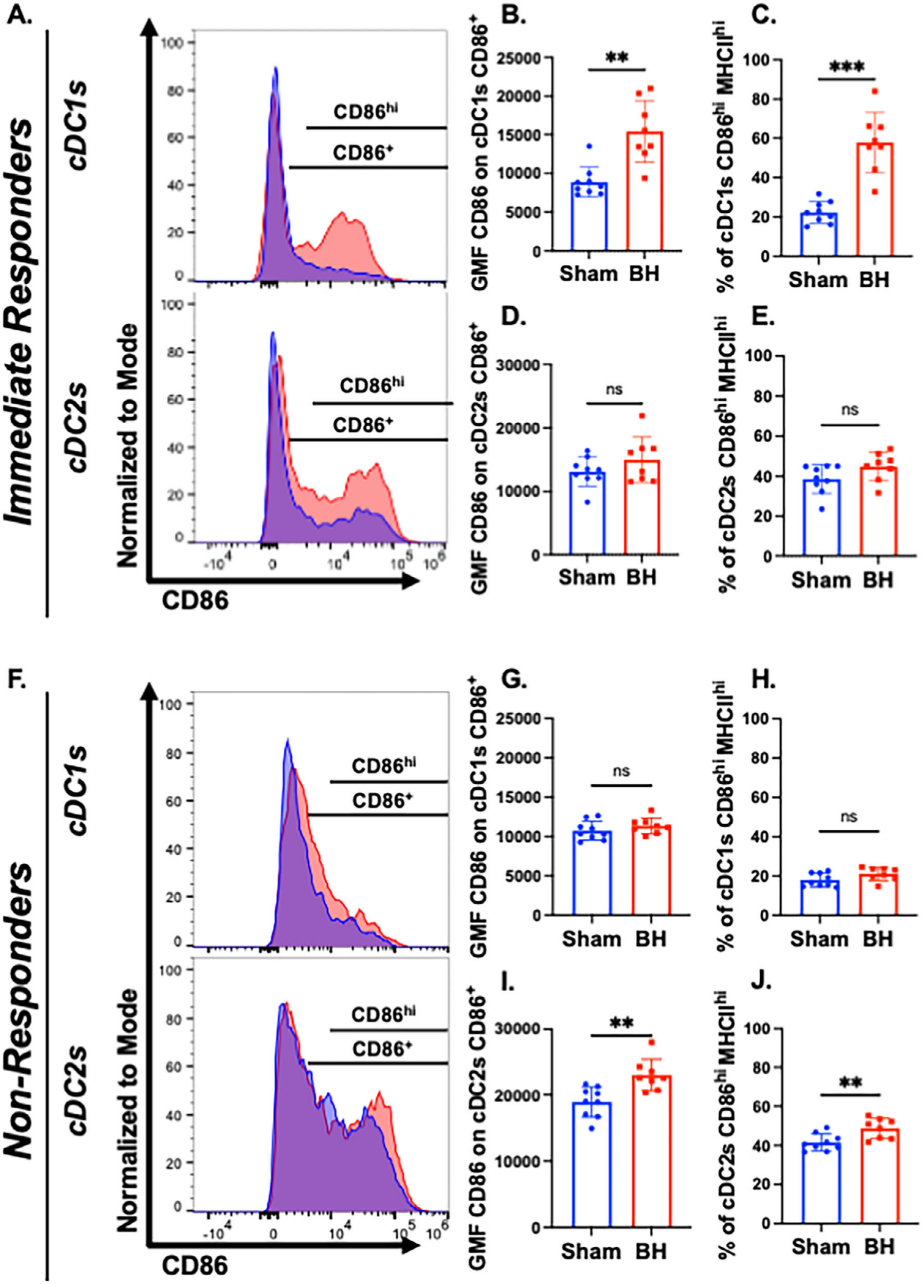
Tumor growth control response by BH is associated with elevated phenotypic activation of cDC1s. (A, F) Histograms showing CD86 intensity on cDC1s (top) and cDC2s (bottom) after treatment with BH or Sham control in the immediate responders (A) and non‐responders (F). (B, G) The intensity of CD86 on total cDC1s CD86^+^ in the TME of immediate responders (B) and non‐responders (G). (C, H) Proportion of cDC1s appearing phenotypically activated (CD86^hi^MHCII^hi^) in the TME of immediate responders (C) and non‐responders (H). (D, I) The intensity of CD86 on cDC2s CD86^+^ in the TME of immediate responders (D) and non‐responders (I). (E, J) Proportion of cDC2s phenotypically activated in the tumors of immediate responders (E) and non‐responders (J). (n = 8‐9) Unpaired t test with Welch's correction: ns = nonsignificant, ∗∗ P<0.01, ∗∗∗ P<0.001; Mean ± SD. ROUT Outliers analysis with Q = 0.1%.

### 2.5 Immediate Response to BH is Associated with an Increased Frequency of CCR7 Expressing cDC1s

Given that cDC migration from the periphery to draining lymph nodes is dependent on the chemotactic axis of the CCR7 receptor and its ligands CCL19 and CCL21 [24, 25], we sought to determine whether BH impacts CCR7 expression on cDCs in the TME. Mirroring the activation status, we found that cDC1s in immediate responders experienced a significant increase in proportion expressing CCR7 acutely post BH, while no alterations were observed on the cDC2 population (Figure 7A–E and Figure S18). In the non‐responders, minimal to no changes were seen (Figure 7F–J). As CCR7 is also upregulated during cDC activation, these data further support that BH treatments resulting in tumor growth control are inducing the activation of cDC1s in the TME.

**FIGURE 7:**
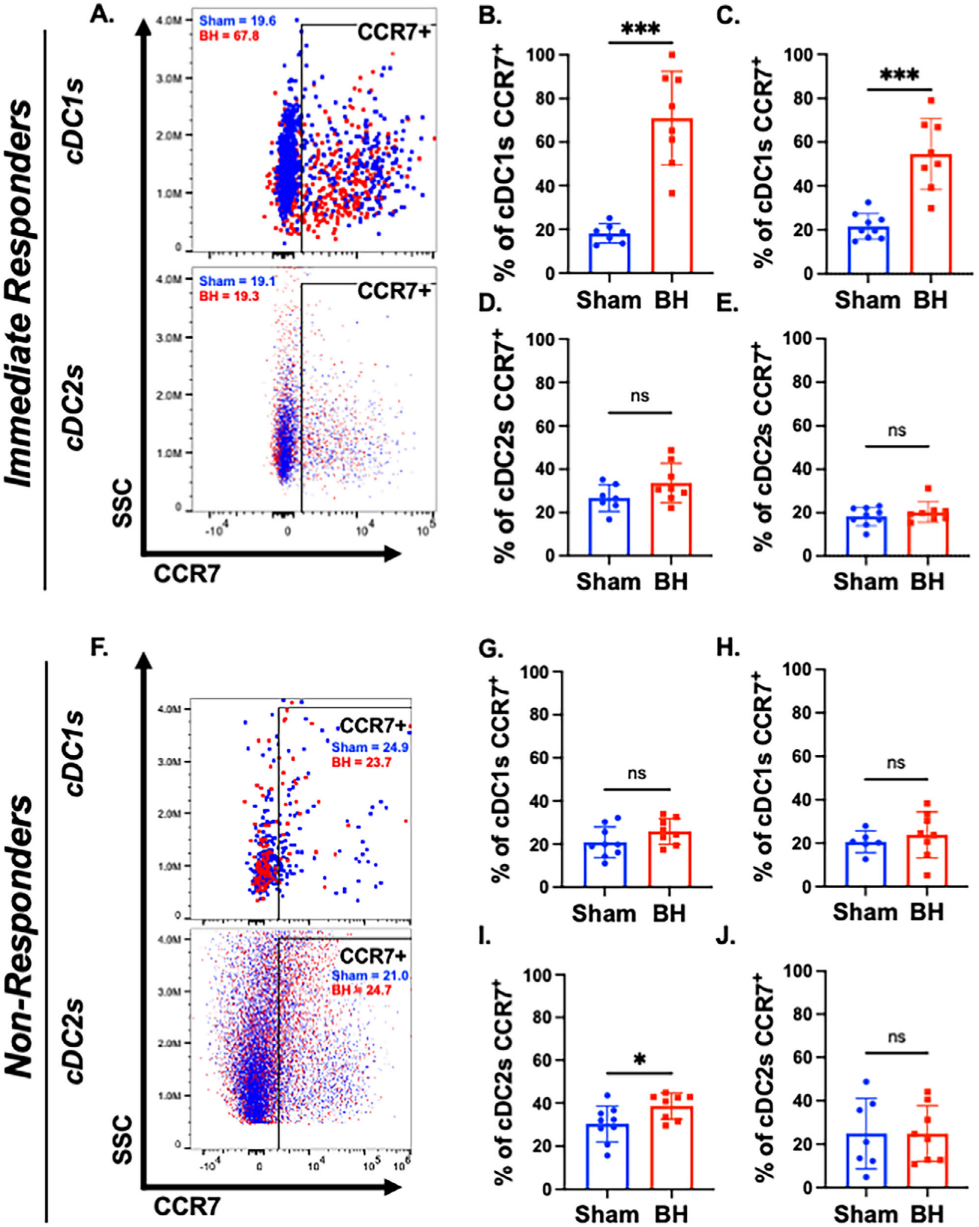
BH‐induced immediate tumor control is associated with an increased frequency of CCR7 expressing cDC1s. (A, F) Scatter plots showing CCR7 frequency on cDC1s (top) and cDC2s (bottom) 24 h after treatment with BH or Sham control in immediate responder (A) or non‐responder tumors (F). (B, C) Proportion of cDC1s CCR7^+^ in the tumors of the immediate responder cohorts. (D, E) Proportion of cDC2s CCR7^+^ in the tumors of the immediate responder cohorts. (G, H) Proportion of cDC1s CCR7^+^ in the tumors of the non‐responder cohorts. (I, J) Proportion of cDC2s CCR7^+^ in the tumors of the non‐responder cohorts. (n = 8‐9) Unpaired *t*‐test with Welch's correction: ns = non‐significant, ∗ *P* < 0.05, ∗∗∗ *P* < 0.001; Mean ± SD. ROUT Outliers analysis with Q = 0.1%.

However, cDC1 activation post BH in the TME of immediate responders in both melanoma models (Figures 6 and 7 and Figures S17 and S18) did not correspond with elevated phenotypic T‐cell activation or expansion in the TME at this acute timepoint. We observed no increase in the proportion of CD8^+^ or CD8^−^ T‐cells that express the canonical T‐cell activation markers CD69 and CD25 between the Sham control and immediate responder tumors (Figures S19A,B and S20A,B). As CD8^+^ T‐cells are the primary cytotoxic cells, we assessed B16‐ZsG immediate responders versus non‐responders by normalizing the percent of CD8^+^ T‐cells that are CD69^+^CD25^+^ in the BH cohorts to that of the average of their respective Sham cohort. We then compared the fold change of the immediate responders to the non‐responders and found that there is no difference between the proportion of CD8^+^ T‐cells expressing activation markers 24 h post BH in the TME (Figure S19C). These data suggest that the CD8^+^ T‐cells present in the TME of immediate responders are not more activated at this timepoint than those detected in the non‐responders. We also examined T‐cell presence and proliferation in the TME 96 h post BH in the immediate responder cohorts and found that there are still significant and strong trends of reduced T‐cell numbers at this later timepoint in both models (Figures S19D,E and S20C,D). There is also no difference in the proportion of cells expressing Ki67 – a hallmark transcription factor for proliferation – between the Sham and BH cohorts (Figures S19F,G and S20E,F).

### 2.6 Cavitation Scores Correlate With ZsG Tumor Antigen Presence in the TDLNs 24 h Post BH Ablation

Utilizing our cavitation scoring metric detailed in the methods section “Cavitation scoring” (Figure S21A,B), we examined whether B‐mode imaging predicts the degree of antigen accumulation in the TDLNs acutely post‐ablation. When we compared the number and proportion of total live immune cells that had acquired ZsG tumor antigen in the TDLNs of the BH cohorts, we found significantly more ZsG present in the TDLNs of immediate responders (Figure 8A,C). Interestingly, the amount of ZsG^+^ immune cells in the TDLNs post BH strongly correlates with their respective cavitation score (Figure 8B,D). In all, these data suggest that higher cavitation scores may reflect differential BH responses that enhance antigen flow through the lymphatics independent of tumor growth control response.

**FIGURE 8:**
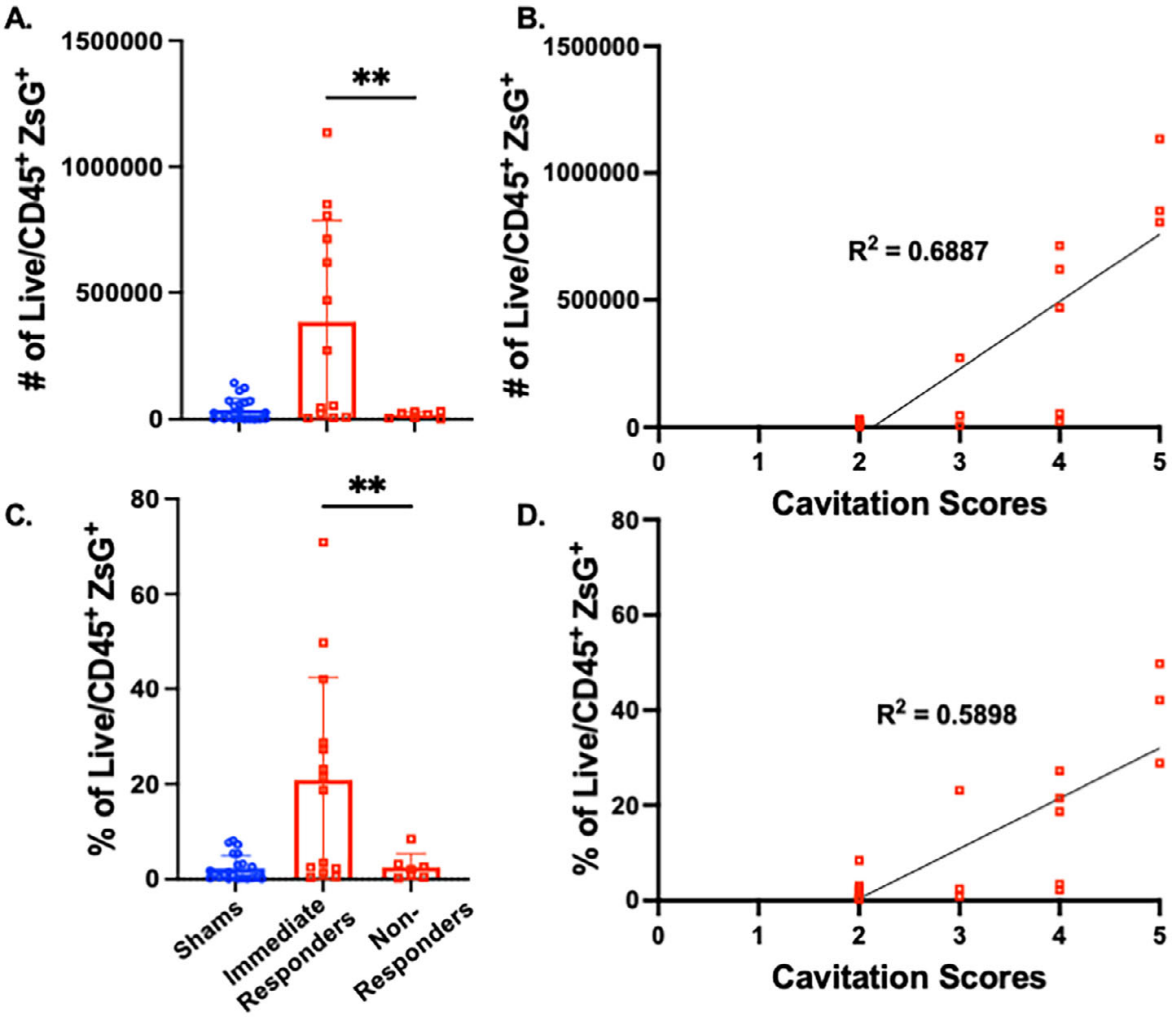
Cavitation scores correlate with ZsG tumor antigen acquisition amongst total live immune cells in the TDLNs 24 h post BH ablation. (A) Number of live CD45^+^ ZsG^+^ cells in the TDLNs of 24 h cohorts from shams, immediate responders and non‐responders (n = 23/shams, n = 15/immediate responders & n = 8/non‐responders). (B) Linear regression correlation between the number of live CD45^+^ ZsG^+^ cells in the TDLNs 24 h post BH and cavitation scores (n = 23). (C) Proportion of live CD45^+^ cells that are ZsG^+^ in the TDLNs of 24 h cohorts from shams, immediate responders and non‐responders (n = 23/shams, n = 15/immediate responders & n = 8/non‐responders). (D) Linear regression correlation between the proportion of live CD45^+^ ZsG^+^ cells in the TDLNs 24 h post BH and cavitation scores (n = 23). Unpaired *t*‐test with Welch's correction: ∗∗ P<0.01; Mean ± SD (A, C). Simple linear regression model (B, D). ROUT Outliers analysis with Q = 0.1%.

### 2.7 Relationships Between Cavitation Score and Tumor Growth Control

While cavitation scoring provided predictive insight into whether BH treatment results in the accumulation of tumor antigen in the TDLNs, we ultimately wanted to test whether this metric could foretell BH‐induced tumor growth constraint. Upon implementation of this scoring regimen into our BH treatments, we observed that higher median cavitation scores on a given treatment day were associated with growth response (Figure S21C–F). We observed a trend in individual growth rates calculated from the day of BH ablations to the 96 h endpoint being higher in the non‐responders (Figure 9A); indicating that the tumors in this setting grew at a faster rate post BH treatment than immediate responders. Additionally, we found that the cavitation scores recorded for immediate responders were significantly higher than for non‐responders (Figure 9B). Porting the response data into a simple logistic regression analysis allowed us to estimate the probability of achieving an immediate responder for a given cavitation score (Figure 9C). The cavitation score yielding a 50% chance of an immediate responder (CS50) was 3.304. The Receiver Operating Curve showing the quality of this logistic regression is provided in Figure 9D. However, when we performed a linear regression analysis of tumor outgrowth rates as a continuous variable post BH versus their respective cavitation scores, we found no significant correlation between these metrics (Figure S21G). Consistent with this result, as assessed by linear regressions, cavitation scores did not correlate with cDC immune responses in the TMEs of immediate responders and non‐responders 24 h post‐ablation (Figure S22A–D). However, they did moderately correlate with macrophage responses at this timepoint (Figure S22E,F), suggesting that cavitation scoring may provide insight into how BH is affecting one of the most abundant cell populations in these TMEs (macrophages), but that this metric is not predictive of how BH is going to affect less prevalent cell subsets such as cDCs.

**FIGURE 9:**
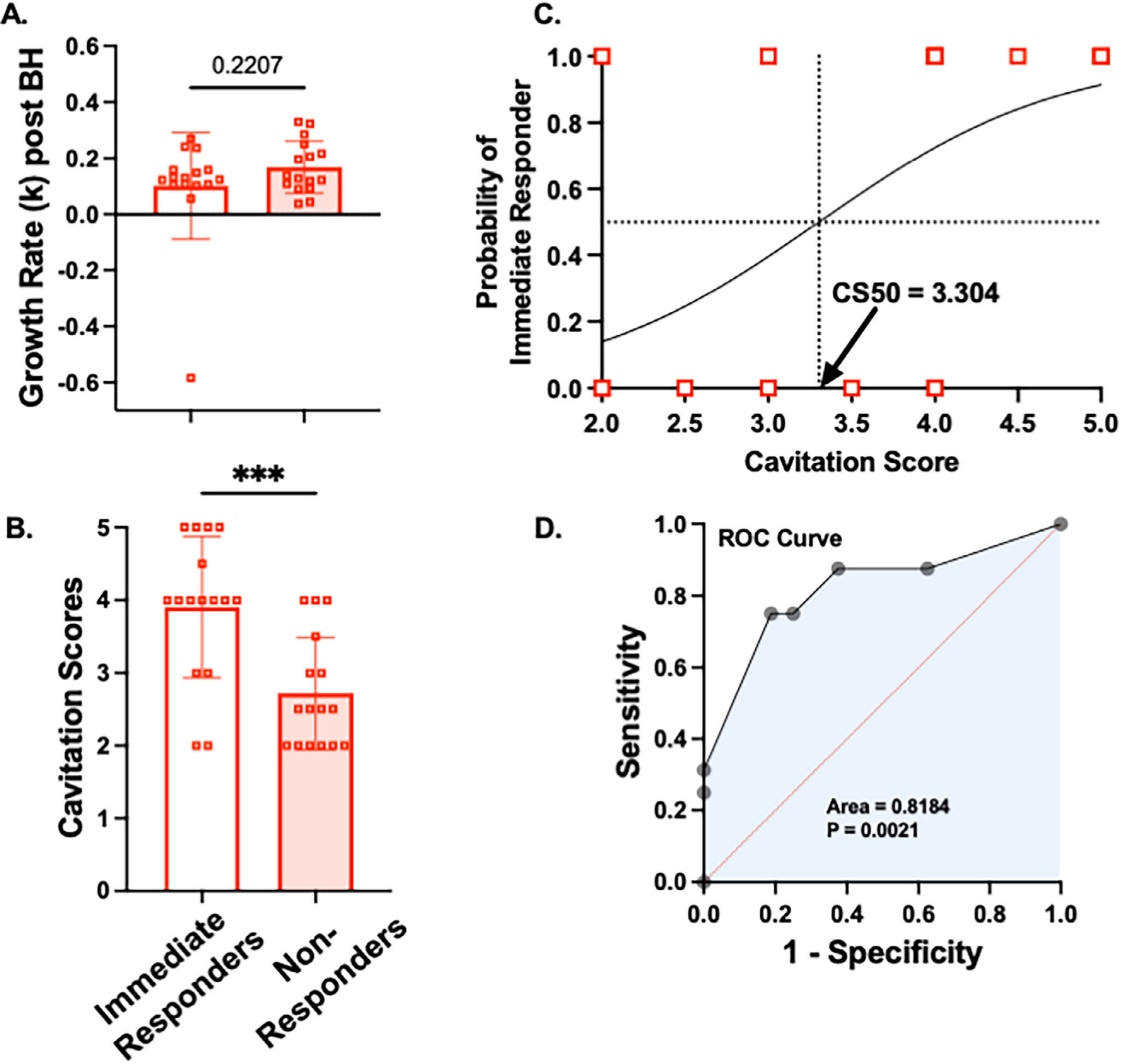
Relationships between Cavitation Score (CS) and tumor outgrowth. (A) Tumor outgrowth rates (k) post BH in the 96 h cohorts from immediate responders and non‐responders. (B) Cavitation scores from treatments of the 96 h BH cohorts from immediate responders and non‐responders (n = 16/group). Unpaired t tests with Welch's correction: ∗∗∗ *P* < 0.001; Mean ± SD. (C) Simple logistic regression showing the probability that a given CS will generate an Immediate Responder. The CS yielding a 50% chance of an Immediate Responder (CS50) was calculated to be 3.304. (D) Receiver operator curve (ROC) for the simple logistic regression in (C) P = 0.0021; Area Under the Curve = 0.8184.

## 3 Discussion

Mechanically fractionating melanoma tumors with BH has the potential to promote T cell‐mediated anti‐tumor responses. A better understanding of the extent to which BH monotherapy drives dendritic cell tumor antigen acquisition and activation in the TME and the identification of BH‐induced tumor outgrowth signatures that associate with immune activation will likely provide insights as to how to optimally leverage immunity to interact with BH. To this end, we addressed gaps in knowledge regarding the impact that BH has on APC presence in the TME, antigen acquisition, and cDC activation states in settings where BH does or does not induce acute primary tumor growth inhibition, independent of BH‐induced bioeffects in the tumor. By utilizing spectral flow cytometric analysis, we found that the immediate (i.e., within 4 days of treatment) degree of BH‐mediated growth inhibition correlates with profoundly divergent consequences on immune cell populations in the TME. When BH treatment acutely impeded melanoma outgrowth, (i) immune cell presence in the TME was significantly reduced, (ii) tumor antigen acquisition amongst APC subsets was elevated when compared to Sham controls, and (iii) intratumoral immune activation was observed distinctly in the cDC1 subset of conventional dendritic cells. In contrast, in the absence of BH‐induced acute growth, there were no differences in the intratumoral immune composition or acquisition of tumor antigen, and only partial cDC activation was observed solely in cDC2s when compared to Sham treated tumors. These striking findings suggest that the degree of immediate BH‐driven tumor growth control positively correlates with signatures of innate immune activation in melanoma tumors that could support T‐cell responses. In turn, immediate growth control after BH may be prognostic for intratumoral immune activation. This information provides rational for fine‐tuning BH regimens to alleviate tumor burden and optimally generate an antitumoral immune response.

It has been well established that the presence of APC subsets in the TME have positive and negative prognostic implications [17, 23]. Here, we determined that when treating melanoma tumors with BH resulted in growth constraint, the number of cDCs residing in the TME was significantly reduced (Figure 2E,I). While we predict this is due to cell death induced by the ablation, and not the result of increased cDC migration to the TDLNs, future studies impeding cDC trafficking are required to make this determination. This effect was not sustained, as the cDC1 and cDC2 populations returned to the Sham baseline by 96 h (Figure 3A,E), indicating that these critical APCs can repopulate the residual tumor. In contrast, when BH did not alter primary tumor outgrowth, there were no alterations in APC presence observed at either the 24 or 96 h timepoints post treatment (Figures 2 and 3). As mature cDCs generally do not proliferate, these populations are most likely rebounding due to the recruitment of circulating precursor‐DCs (pre‐cDCs). pre‐cDCs can be found in both the bone marrow and blood, and they rely on chemotactic gradients to extravasate out to the TME. Pre‐cDC1s rely on the CXCR3‐CXCL9/10 axis [29], and cDC1 accumulation in tumors requires the XCR1‐XCL1 axis [30]. cDC2s utilize the CCR2‐CCL2 axis [31]. Interestingly, the number of F4/80^+^ macrophages was not altered by BH ablation (Figure 2M). This could be the result of macrophages repopulating the TME at a faster rate than cDCs following insult to the tumor via proliferation of residual cells remaining post ablation and/or recruitment of precursor circulating monocytes that then differentiate into macrophages in the TME [32]. It is also possible that macrophages may be less sensitive to mechanical stress than cDCs. Some studies indicate that cDC1s are required for tumor‐specific CD8^+^ T‐cell recruitment into the TME [33] and that cDCs in the TME can provide a secondary co‐stimulation signal via CD80/CD86 to promote CD8^+^ T‐cell effector function [18]. To be able to achieve the secondary co‐stimulation and promote T‐cell recruitment, the cDCs must be activated. However, cDCs retained in tumors can develop an unresponsive phenotype transcriptionally, where they downregulate genes associated with antigen presentation and activation [34]. Therefore, it is possible that BH may deplete dysfunctional or tolerogenic cDC populations within the TME, thereby permitting the influx of more functional cDCs. However, this remains speculative and will require direct investigation in future studies.

We also provide evidence that tumor antigen is not only liberated by BH ablation, but that this antigen is being acquired at an enhanced level by the cDC populations in the TME. Similar to the effects on APC abundance, we determined that acute tumor growth response to BH corresponds with increased capture of tumor antigen by APCs. In the context of immediate growth control, BH significantly raises the proportion of APCs that acquire tumor antigen (ZsG) in the TME within 24 h post‐ablation (Figure 4A–D and Figure S14B–E). Most of these effects were sustained through 96 h (Figure 5A–C and Figure S14F–H). As no differences were observed in tumor antigen acquisition by intratumoral APCs in the non‐responsive tumors (Figure 4E–H), the increases in ZsG acquisition appear associated with BH‐mediated tumor control. The use of ZsG as a model tumor antigen is a potential limitation of this study. While ZsG fluorescence enables sensitive single‐cell tracking of antigen uptake, it does not directly report on endogenous peptide processing and MHC presentation. However, prior work has shown that ZsG‐containing vesicles in tumor‐associated APCs co‐localize extensively with canonical melanoma antigens, indicating that ZsG traffics within shared antigen‐processing compartments [35]. In this context, we believe that ZsG serves as a proxy for bulk tumor antigen acquisition. Future studies incorporating endogenous antigen‐specific readouts (e.g., peptide–MHC complexes or tumor‐specific T cell receptor‐based assays) will be important to further validate functional antigen presentation following BH.

Another objective of our work was to interrogate whether BH, in either the setting of eliciting growth inhibition or not, provides an inflammatory signal that activates cDC populations. cDC1s are the canonical APC capable of directly cross‐presenting antigen to CD8^+^ T‐cells and eliciting a potent anti‐tumor immune response. When cDC1s expand, accumulate and become activated at the site of the tumor, therapeutic responsiveness of melanoma to ICB and targeted therapies is improved [36]. We found that in the setting of immediate tumor control, CD86 upregulation was promoted in the residual intratumoral cDC1 population (Figure 6A–C and Figure S17A–C), while in the absence of acute tumor control, CD86 upregulation was only observed in the cDC2 population (Figure 6F–J). It is of note that while no differences in activation were observed in the cDC2 population from B16‐ZsG tumors (Figure 6A,D,E), cDC2s from BH‐inhibited YUMM‐ZsG tumors displayed higher levels of activation 24 h post treatment (Figure S17A,D,E). In addition to these melanoma models having different degrees of tumor mutational burden, they have also been shown to have different tumor growth rate kinetics, responsiveness to chemotherapeutic agents, antigen‐specific T‐cell responses, and immune infiltration (Figure S23) [37]; all of which may play a role in any divergence observed between how cDCs in these two models respond to BH. Moreover, we saw in both murine melanoma models that when BH impeded tumor outgrowth, there was a significant increase in the proportion of cDC1s that expressed CCR7 (Figure 7A–C and Figure S18B,C), which is upregulated upon cDC maturation and activation [38, 39]. cDC activation can occur in response to the release of immune stimulatory damage‐associated molecular patterns (DAMPs), through the activation of natural killer cells, or through a reduction in immunosuppression, such as decreased inhibitory cytokine or receptor expression [40]. While we cannot distinguish the individual roles of these pathways for cDC activation in these studies, we have observed that cDC activation only occurs in settings where melanoma outgrowth is delayed. These results provide insight into a potential mode of BH‐mediated antitumoral immunity with the level of growth constraint corresponding to cDC1 activation.

It should be noted a cytolytic CD8^+^ T‐cell response takes on the order of ∼7 days to occur [41] and our findings focus on alterations to tumor outgrowth within 96 h (4 days) post BH ablation, we are not attributing the growth constraint observed in the immediate responder cohorts to the cDC1:CD8^+^ T‐cell axis. Rather we propose that the immediate responder phenotype and the allied antigen liberation and alterations in immune cell presence observed acutely are associated with physical tumor debulking. Importantly, while these findings do not support a direct role for early T‐cell‐mediated tumor control, they do establish a mechanistic framework in which BH‐induced antigen release and cDC1 activation may serve as initiating events that prime subsequent adaptive immune responses at later timepoints, motivating future long‐term outgrowth and survival studies.

As a monotherapy, BH is not curative in pre‐clinical melanoma models. However, when ICB was used in the adjuvant setting of HER2‐expressing breast cancer and in the neoadjuvant setting of B16 melanoma expressing the exogenous LCMV antigen gp33, the efficacy of mechanically ablative FUS to alleviate distal tumor‐burden was enhanced [11, 12]. Nevertheless, these results did not yield complete responders. We believe the positive immunological effects observed when BH induces the responder phenotype can be leveraged to improve therapeutic efficacy to immune‐stimulating agents, such as ICB and toll‐like receptor (TLR) agonists. When the cDC1 population is activated at the tumor site, therapeutic responsiveness of melanoma to ICB and targeted therapies is improved [36]. Here, cDC1s were activated acutely by BH in the responder setting (Figures 6 and 7; Figures S17 and S18). However, cDC1 activation post BH in the melanoma TME of immediate responders does not correspond with elevated phenotypic T‐cell activation or expansion during this acute timepoint analysis (Figures S19 and S20). This suggests that a more potent stimulation and stronger cDC interaction may be required to induce acute T‐cell activation. Even though BH increases the activation status of cDCs, this activation and upregulation of CD86 is to a much lesser degree than that induced by the TLR stimulant polyI:CLC – a mimic of the DAMP double‐stranded RNA [10]. Therefore, a rational therapeutic combination would be to (1) employ BH to physically debulk the tumor and increase tumor antigen acquisition by cDCs, (2) administer intratumoral polyI:CLC to further activate tumoral cDCs and promote cDC migration to the TDLNs, and (3) administer systemic ICB to alleviate T‐cell immunosuppression so that their cytolytic capabilities are not dampened. Future studies are required to determine the effect of treatment sequencing and timing (i.e., neoadjuvant, adjuvant, or concurrent) on therapeutic efficacy, tumor antigen release, cDC activation, and downstream T‐cell responses to optimize the integration of BH into clinically relevant treatment regimens.

In addition, cavitation scoring, a qualitative diagnostic ultrasound metric based on real‐time B‐mode imaging signatures characteristic of BH‐induced tissue destruction, correlates with ZsG tumor antigen presence in the TDLNs 24 h post treatment (Figure 8). These findings provide clarity in that antigen liberated through the mechanical ablation of melanoma tumors is not being sequestered from cDCs by another non‐professional APC in the TME, and that cDCs remaining in the TME as well as those repopulating the TME during the 96 h span post BH can effectively engulf tumor antigen – a crucial first step required for cross‐presentation. To our knowledge, cavitation scoring is the first metric to provide predictive insight into whether BH treatment will result in acute tumor antigen dissemination to the TDLNs. However, while cavitation scoring is a predictor of antigen delivery to the TDLNs, it is not predictive of tumor growth rate when considered as a continuous variable (Figure S21G), as opposed to the discrete immediate responder/non‐responder classification. We also observe variable correlation between cavitation scoring and APC presence and acquisition of tumor antigen (Figure S22). It is only when these studies are examined as entire cohorts, using the discrete responder vs. non‐responder classification, that we observe higher cavitation scores being associated with the responders and their subsequent lower outgrowth rates (Figure 1, Figure 9A,B and Figure S21C–F). One possibility for this may be that cavitation scoring is not a nuanced or inherently quantitative metric. For example, it may be able to provide insight into how BH is affecting one of the most abundant cell populations in these TMEs (macrophages), but it is unable to predict how BH is going to affect less prevalent cell subsets such as cDCs. Additionally, while cavitation scoring is based on visual ultrasound signals characteristic of BH‐like fractionation, it is not an absolute metric of tissue destruction. During the BH ablations, any external bubble or acoustic noise will be visible on ultrasound imaging. These artifacts not associated with the treatment itself can appear as a hyperechoic mark on imaging and may explain why cavitation scores do not correlate with a reduction in tumor outgrowth rates on an individual level.

A key aspect of this study is the use of post‐treatment tumor growth kinetics to define response. While we demonstrate that early changes in tumor growth rate on average strongly associate with intratumoral immune composition and response, we acknowledge that the use of a fixed threshold to define “responders” is inherently operational and not universally standardized. This challenge is not unique to our study. Increasingly, both preclinical and clinical oncology studies recognize that tumor response exists along a continuum, and that traditional categorical metrics may fail to capture biologically meaningful treatment effects. In this context, our use of growth rate serves as a biologically informed stratification tool rather than a definitive classification scheme. The fact that this approach delineates groups with markedly distinct immune phenotypes supports its relevance in the present system. Nevertheless, future work incorporating model‐based or data‐driven classification of tumor growth trajectories may further refine response definitions and improve generalizability across studies [42, 43].

Notably, we observed divergent tumor growth responses despite the use of identical BH parameters (Figure 1). This variability likely arises from a combination of physical and biological heterogeneity. Differences in tumor acoustic properties, vascularization, and tissue architecture may influence cavitation dynamics and the degree of mechanical fractionation achieved [44, 45, 46]. In parallel, variability in the TME, including immune composition, stromal content, lymphatic composition, and perfusion may modulate the downstream biological response to BH. These findings highlight the importance of integrating both biophysical and biological context when interpreting response to mechanical ablation and underscore the need for future studies that incorporate real‐time monitoring and predictive modeling to better understand and control treatment variability. Furthermore, these studies focused on the effects of a single BH treatment. Tumor growth and immunological responses to subsequent BH ablations could be vastly different due to the altered physical and biological composition of the TME following initial treatment. Future work is required to evaluate how repetitive BH treatments influence the physical ablation process and downstream immune responses.

There are important limitations to the current study. As we have not examined the ensuing consequences of cDC antigen acquisition and activation, these data do not establish that cDC1 activation is required for acute tumor growth control or that cDC1s directly promote T‐cell responses at these early timepoints. Future functional studies utilizing cDC1‐deficient models such as *Batf3^−/−^* or Irf8+32^−/−^ [47, 48], CD8^+^ T‐cell depletions, and assays of tumor‐associated antigen‐specific T‐cell cytotoxicity will be required to determine whether the observed cDC1 phenotype contributes causally to the growth inhibition present in the immediate responder cohorts and/or durable antitumor immunity. Similarly, T‐cell analyses were limited to abundance, early activation markers, and proliferation, and did not directly measure effector functions such as IFN‐γ, TNF‐α, Granzyme B, or cytolytic activity of endogenous or antigen‐specific T‐cell responses; all of which would be pertinent for establishing whether BH in either the responder or non‐responder setting is eliciting cytolytic CD8^+^ T‐cell or helper CD4^+^ T‐cell activation against these melanoma tumors. Second, while ZsG enabled sensitive single‐cell tracking of tumor antigen dissemination following BH, ZsG positivity should be interpreted as a readout of bulk antigen acquisition rather than direct evidence of antigen processing, peptide–MHC loading or functional antigen presentation to T‐cells. Third, although APC reductions in immediate responder tumors are consistent with BH‐induced tissue destruction, the mechanism underlying reduced cDC abundance remains unresolved. Cell death was not directly measured within the ablated tumor, and we have not impeded cDC migration to determine the contribution of cDC trafficking on the observed APC reduction. Fourth, because our analyses relied primarily on flow cytometry data from dissociated tumors, they do not preserve spatial information regarding whether APC subsets localize within ablated, peri‐ablative, or viable tumor regions [49]. Future studies examining the location of APCs and other immune populations are required to determine whether BH augments the dispersion of immune cells in these tumors. Finally, the molecular signals responsible for BH‐associated APC activation were not directly elucidated in this study. Therefore, potential contributions of DAMP release, cytokine/chemokine signaling, innate immune sensing pathways, or altered immunosuppression remain speculative. Taken together, these findings should be interpreted as identifying acute immunologic correlates and prognostic features of productive BH‐mediated tumor outgrowth disruption, rather than establishing a complete causal mechanism linking BH, cDC1 activation, antigen presentation, and adaptive immune‐mediated tumor control.

## 4 Materials and Methods

### 4.1 Mice

All mouse experiments were conducted in accordance with the guidelines and regulations of the University of Virginia and approved by the University of Virginia Animal Care and Use Committee. All mice were obtained from The Jackson Laboratory (Jax, Bar Harbor, ME, USA). Eight‐ to ten‐week‐old male C57Bl/6J mice were used for the melanoma studies (Jax #000664). All mice were housed on a 12 h/12 h light/dark cycle and supplied food ad libitum.

### 4.2 Tumor Models and Implantation

The stably transduced B16F10‐ZsGreen cell line was a gift from Dr. Matthew Krummel at the University of California, San Francisco [25], and they were maintained in RPMI‐1640+L‐Glutamine supplemented with 5% fetal bovine serum (FBS, Corning (Glendale, AZ, USA) #35‐010‐CV). The YUMM1.7 cell line was gifted to us by Dr. Drew Dudley's lab at the University of Virginia. Utilizing a lentivirus containing ZsGreen (ZsG), we transduced the YUMM1.7 cells to stably express the fluorescent ZsG antigen. The ZsG^+^ cells were enriched via sorting on the Influx Sorter in the UVA Flow Cytometry Core and expanded. These cells were grown in DMEM/F12 (Gibco #11330‐032) supplemented with 10% FBS and 1% Non‐Essential Amino Acids (Gibco #11140‐050). All cells were grown at 37°C with 5% CO_2_. Thawed cells were cultured for up to three passages for all experiments. Cells tested negative for mycoplasma prior to freezing.

3 × 10^5^ melanoma cells (B16‐ZsG or YUMM‐ZsG) were subcutaneously (s.c.) implanted into the shaved right flank of mice through a 25G × 1 ½ in needle (BD PrecisionGlide Needle #305127). Tumor outgrowth was monitored via digital caliper measurements. Tumor volume was calculated as follows: volume = (length × width^2^)/2. On the day of treatment, mice were randomized into cohorts in a manner that ensured matching of mean starting tumor volumes across experimental groups.

### 4.3 In Vivo Ultrasound‐guided Boiling Histotripsy

Melanoma tumor‐bearing mice underwent Sham or BH treatment 13 days post tumor inoculation. On treatment day, mice were anesthetized with an i.p. injection of ketamine hydrochloride injection (20 mg/mL; Zoetis) and dexmedetomidine hydrochloride (0.05 mg/mL; Dechra) in sterilized 0.9% saline (Hospira #PAA128035). Dexmedetomidine hydrochloride was reversed with a i.p. injection of atipamezole hydrochloride (Revertidine, Modern Veterinary Therapeutics) after Sham or BH treatment. Right flanks of mice were reshaved to remove hair that had regrown since tumor implantation, after which BH was performed using an inhouse built system. The imaging transducer – an Acuson S2000 Helix Evolution Touch, 14L5 SP imaging probe, 10 MHz, 25 mm field width linear imaging array (Siemens, Inc.) – is orthogonally coupled to a 2.5 MHz center‐frequency, single‐element therapy transducer. An arbitrary function generator (Tektronix, AFG 3052C) and amplifier (E&I, 1040L) were used in conjunction with the therapy transducer to produce the BH treatments. Both the imaging and treatment transducers were ultrasonically coupled to the animal using degassed, deionized water at 37°C during the duration of each BH treatment. BH was applied in a pulsed fashion for 10 s, at a peak negative pressure = 21 MPa, pulse repetition frequency = 4 Hz, pulse length = 3 ms, with treatment points spaced 1 mm in a rectangular grid pattern and two planes of treatment, which were separated by 2 mm. With this ablation pattern and focal size, we calculate that ∼20% of each tumor was exposed to BH. The treatment scheme is outlined in Figure 1A. Sham treatment comprised of fully submerging the flank tumor in the 37°C water bath for 6 min.

### 4.4 Immediate Responder Versus Non‐Responder Criterion

Tumor growth curves from day 13 (treatment day) through day 17 (96 h post treatment) were fit using an exponential growth model of the form:

(1) $$V\;\left(t\right)=V_{0}\;e^{kt}$$

where *V*_0_ represents the initial tumor volume and *k* represents the tumor growth rate constant. Growth rate constants were determined for each treatment cohort within an independent experiment. Response classification was performed at the level of the experiment rather than individual tumors. Experiments in which the BH‐treated cohort exhibited a statistically reduced growth rate constant relative to the matched Sham cohort (α < 0.1) were classified as “immediate responders,” whereas experiments not meeting this criterion were classified as “non‐responders.” Parallel 24 h cohorts were retrospectively assigned based on the classification outcome of the corresponding 96 h cohort from the same experiment.

### 4.5 Preparation of Single Cell Suspensions Performed 24 or 96 h Post Treatment

#### 4.5.1 Tumors

Tumors were harvested and enzymatically digested for 1 h at 37°C in RMPI media supplemented with 5% FBS, 20 U/mL Type I Collagenase (Gibco; Grand Island, NY, USA #17018029) and 0.1 mg/mL DNase I (Roche; Branchburg, NJ, USA #10104159001). After digestion, tumors were subjected to manual homogenization (Tenbroeck Tissue Grinder #62400‐518 Wheaton; Ottawa, ON, CAN) and filtered through 100 µm filter mesh (Genesee Scientific; Research Triangle Park, NC, USA #57‐103) to generate single‐cell suspensions, which were then spun down at 1200 RPM for 5 min (Eppendorf 5180; Enfield, CT, USA). Tumor pellets were resuspended in 10 mL of 1× PBS, and then 10 mL of Lympholyte (Cedarlane Labs; Burlington, ON, CAN #CL5035) was underlaid. Tumor samples were centrifuged in the Eppendorf 5180 at 1000 RCF for 20 min with no brake and low acceleration to separate tumor cells from lymphocytes and other immune cells. The layers above the tumor pellet were collected and placed into a clean 50 mL conical tube. 1× PBS was added to fill the conical tube to the 50 mL line. Tumor samples were spun down for 10 min at 800 RCF to pellet cells. Afterwards, the supernatants were decanted, and the pelleted cells were vortexed and resuspended in the ∼200 µL1× PBS remaining in the conical tube. These cells were then transferred to a 96 well V‐bottom plate for staining.

#### 4.5.2 Lymph Nodes

Right axillary and brachial lymph nodes were pooled for tumor‐draining lymph node (TDLN) analysis. Left axillary and brachial lymph nodes were pooled for contralateral (cLN) analysis. LNs were subjected to manual homogenization and filtered through 100 µm filter mesh to generate single‐cell suspensions. These were then spun down at 1200 RCF for 5 min, supernatants were decanted, and all the cells were transferred to a 96 well V‐bottom plate for staining.

### 4.6 Flow Cytometry

Once all samples were transferred to a 96 well V‐bottom plate, they underwent an initial wash with 1X PBS, and then cells were stained for viability using Fixable Live/Dead Blue for 30 min at 4°C. Next, the samples were incubated with anti‐mouse 5 µg/mL anti‐CD16/32 in FACS + 2% NMS (normal mouse serum; Valley Biomedical, Inc., Winchester, VA, USA, # AS3054) to block Fc gamma receptors for 15 min at 4°C. Cells were spun down post Fc block and resuspended in 100 uL of anti‐CCR7 antibody made up 1:100 in FACS + 2% NMS, and stained for 30 min at 37°C. Afterwards, cells were washed with FACS buffer; spun down; resuspended in a mixture of Brilliant Stain Buffer and FACS + 2% NMS at a ratio of 1:9, respectively; and stained for 30 min at 4°C with fluorescent monoclonal antibodies for surface markers: CD45, CD11b, F4/80, CD11c, MHCII, CD3, CD8α, XCR1, CD86, CD19 & Ly‐6G. Antibody clone information, supplier name, and catalog number can be found in Table S1. Following a wash with FACs buffer, the eBioscience FOXp3/Transcription Factor Staining Buffer Set (#00‐5523‐00) was used for intranuclear staining. Cells were resuspended in 100 µL of solution 3 parts Fix/Perm Diluent + 1 part Fix/Perm Concentrate for 55 min at 4°C. Then, the samples were stained for intranuclear factor Ki67. Anti‐Ki67 was made up in 1X Perm/Wash Buffer, and cells were incubated at 4°C for 25 min. Following a wash with 1X Perm Buffer, the cells were lastly fixed in 1X BD FACS Lysis in the dark for 10 min at room temperature. Following a wash with FACS buffer, the cells were lastly fixed in 1X BD FACS Lysis in the dark for 10 min at room temperature. They were then resuspended in FACS buffer for running on the cytometer. Flow cytometry was performed with the Cytek Aurora Borealis (Cytek Biosciences; Fremont, CA, USA) and SpectroFlo v3.0.3 software (Cytek Biosciences). Data were analyzed using FlowJo 10 software (FlowJo, LLC; Ashland, OR, USA). All gating strategies can be found in Figures S2, S4, and S6.

### 4.7 ImageStreamX System – Imaging Flow Cytometer

Similar to conventional flow cytometry, single cell suspensions were transferred to a 96 well V‐bottom plate and underwent an initial wash with 1X PBS. The cells were then stained for viability using Fixable Live/Dead Aqua for 30 min at 4°C. Next, the samples were incubated with anti‐mouse 5 µg/mL anti‐CD16/32 in FACS + 2% NMS to block Fc gamma receptors for 15 min at 4°C. Cells were spun down post Fc block and washed with FACS buffer. They were then resuspended and stained for 30 min at 4°C with fluorescent monoclonal antibodies for surface markers: CD45.2, CD8α or CD11b. Antibody clone information, supplier name, and catalog number can be found in Table S1. Following a wash with FACS buffer, the cells were lastly fixed in 1X BD FACS Lysis in the dark for 10 min at room temperature. They were then resuspended in FACS buffer for running on the Amnis ImageStrreamX Mark II (Cytek Biosciences; Fremont, CA, USA).

### 4.8 ZsGreen Analysis in Murine Melanoma Tumors

We found that analysis of tumor samples with the ZsG^+^ gating strategy previously used to define antigen presence in the LNs, which is based on LNs from a naïve, non‐tumor‐bearing mouse, resulted in the majority of all Live/CD45^+^ cells, including non‐phagocytic CD8^+^ T cells, being deemed positive (Figure S12A). To better refine our gating strategy, we chose to utilize a biological negative control – CD8^+^ T‐cells – as CD8^+^ T‐cells are not phagocytic and should not have engulfed the ZsG tumor antigen. Upon further analysis, it became apparent that each individual tumor sample – in both the Sham and BH cohorts – contained a unique shift in ZsG intensity based on tumor size (Figure S12B). Therefore, an individual gate was drawn on the tumor‐resident CD8^+^ T‐cells for each sample, and this gate served as an internal control for cell subsets analyzed within that particular tumor (Figure S12B,C). It is of note that the ZsG^+^ gate drawn on the CD8^+^ T‐cells is done so using contour plots closest to the majority of the population. This is due to some samples experiencing a spread in ZsG staining on the CD8^+^ T‐cell population, which can be seen in Figure S12B with outliers falling in the ZsG^+^ gate even though this population should be entirely biologically negative. We confirmed by ImageStream analysis that the ZsG signal from the CD8^+^ cell population of B16‐ZsG tumors was not an artifact of our flow cytometry analysis. ZsG fluorescence was present in a distinct punctate pattern on CD8^+^ cells, with limited side scatter (SSC) complexity (Figure S13A); suggesting that fragmented pieces of fluorescent antigen stuck to the surface of these cells – most likely a result of processing and the release of tumor antigens post cell lysis. This ZsG composition was dissimilar to that observed from the CD11b^+^ cells in these tumors. CD11b^+^ cells displayed a diffuse presence of ZsG throughout the entire cell consistent with phagolysosomes, with more visible and complex SSC – indicative of granules within the cells – as well as noticeable blebbing structures in the brightfield channels (Figure S13B). Not surprisingly, the increased intensity of ZsG correlates with the physical size of the tumor (Figure S12D) – indicating that the more ZsG‐expressing tumor cells present at the time of single cell suspension generation contributes to the intensity of ZsG observed in each sample. Together, these data suggests that utilizing CD8^+^ T‐cells as a biological control for our ZsG tumor antigen acquiring gate is sufficient to define antigen acquisition by APCs.

### 4.9 Cavitation Scoring

We developed an observational‐based metric intended to qualify and quantify the efficacy of BH treatments in real‐time to provide insight into BH‐mediated growth alterations and subsequent immune effects. This metric uses diagnostic ultrasound that reflects both the real‐time hyperechoic activity observed during treatment as well as the resulting post‐treatment appearance (hypoechoic) via B‐mode imaging which is used for concurrent focal targeting within the tumor. We have termed this metric “Cavitation Scoring.” Specifically, scores of **1–3 **correspond to the **degree and scale of hyperechoic signatures observed during treatment,** which corresponds to transient cavitation and the onset of boiling activity within the focal zone. Scores of **4–5** represent **more intense and spatially extensive hyperechoic activity during treatment,** combined with **persistent hypoechoic regions visible post‐treatment** on B‐mode imaging. A score of 4 is given when the tumor displays a bright signature during and then a darker signature post treatment, while a 5 is given when we observe a ‘blender’ effect—which is the combination of **extensive hyperechoic and persistent hypoechoic regions** happening in real‐time resulting in the visible movement of liquified tissue on B‐mode imaging (Figure S21A,B). Overall, these signatures are characteristic of complete BH‐like fractionation and theoretically should correlate well with localized tissue homogenization.

### 4.10 Statistical Analysis

Statistical analyses were performed in GraphPad Prism 9 (GraphPad Software). A mixed‐effects model with multiple comparisons comparing each cell mean with every other cell mean on that row was run on average tumor outgrowth curves to assess statistical differences in BH versus Sham tumor outgrowth on day 17 – 96 h post BH ablation. ROUT outliers’ analysis with Q = 0.1% was ran prior to any flow cytometry analysis. When comparing two groups of data at a single timepoint, an unpaired, two‐tailed *t*‐test with Welch's correction (i.e., did not assume equal standard deviations) was performed. To assess correlations, a simple linear regression model was used. The probability of treatment resulting in an immediate response was generated using a simple logistics model. All figures, unless otherwise stated in the figure legend, show the mean ± standard deviation (SD). *p*‐values and significance are specified in figure legends. Graphical renderings were made with BioRender.com.

## Author Contributions

**Lydia E Kitelinger**: conceptualization, investigation, methodology, data curation, visualization, project administration, writing – original draft preparation, review and editing. **Matthew R DeWitt**: conceptulization, investigation, methodology, writing – original draft preparation, review and editing. **Carly M Van Wagoner**: investigation, methodology, writing – review and editing. **Claire A Conarroe**: investigation, methodology, writing – review and editing. **Charles C Funk**: investigation, methodology, writing – review and editing. **Aaron B Streit**: investigation, methof = dology, writing – review and editing. **AeRyon Kim**: investigation, methodology, writing – review and editing. **Richard J Price**: conceptualization, methodology, data curation, resources, project administration, writing – review and editing. **Timothy N J Bullock**: conceptualization, methodology, resources, project administration, writing – review and editing. All authors have read and agreed to the pubished version of the manuscript.

## Funding

This work was supported by the Focused Ultrasound Foundation (Boiling histotripsy for the treatment of melanoma – AC10266); NIH R01EB030007 to RJP and TNJB; NIH R21CA286367 and R01CA279134 and grants from the University of Virginia's Focused Ultrasound Immuno‐Oncology (FUSION) and Comprehensive Cancer Centers to RJP.

## Conflicts of Interest

The authors declare no conflicts of interest.

## Supporting information




**Supporting File**: advs76722‐sup‐0001‐SuppMat.docx.

## Data Availability

The data that support the findings of this study are available from the corresponding author upon reasonable request.
