# Supplementary material for: Immediate Growth Control in Response to Boiling Histotripsy is Prognostic for Intratumoral Immune Activation
Source: Adv Sci (Weinh). 2026 Jul 24:e76722. Online ahead of print. doi: 10.1002/advs.76722 (PMC13398126; doi:10.1002/advs.76722)
Supplement: Supplementary file 1 — Supporting File: advs76722‐sup‐0001‐SuppMat.docx. [file ADVS-9999-e76722-s001.docx]

Kitelinger et al., Supplementary Figures


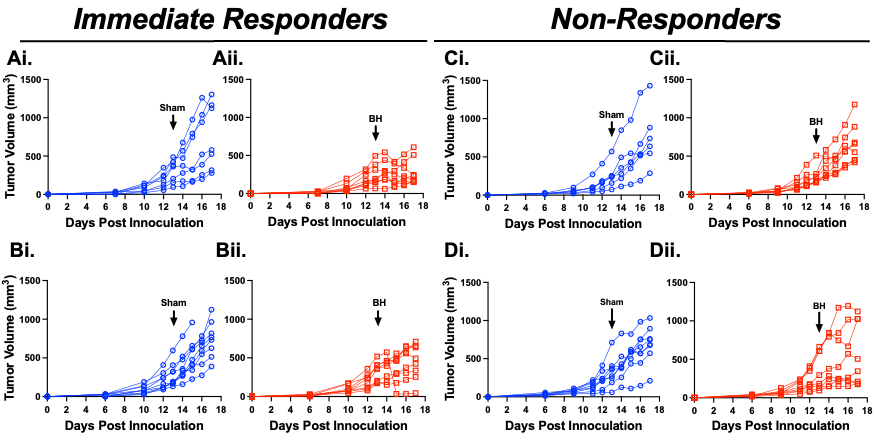


**Supplemental Figure 1. BH treatment protocol yields a bifurcating primary tumor growth control response in subcutaneous melanoma. A.** Individual 96 h cohort tumor growth curves of Sham (**Ai**) or BH (**Aii**) treated mice from Figure 1B. **B.** Individual 96 h cohort tumor growth curves of Sham (**Bi**) or BH (**Bii**) treated mice from Figure 1C. **C.** Individual 96 h cohort tumor growth curves of Sham (**Ci**) or BH (**Cii**) treated mice from Figure 1D. **D.** Individual 96 h cohort tumor growth curves of Sham (**Di**) or BH (**Dii**) treated mice from Figure 1E.


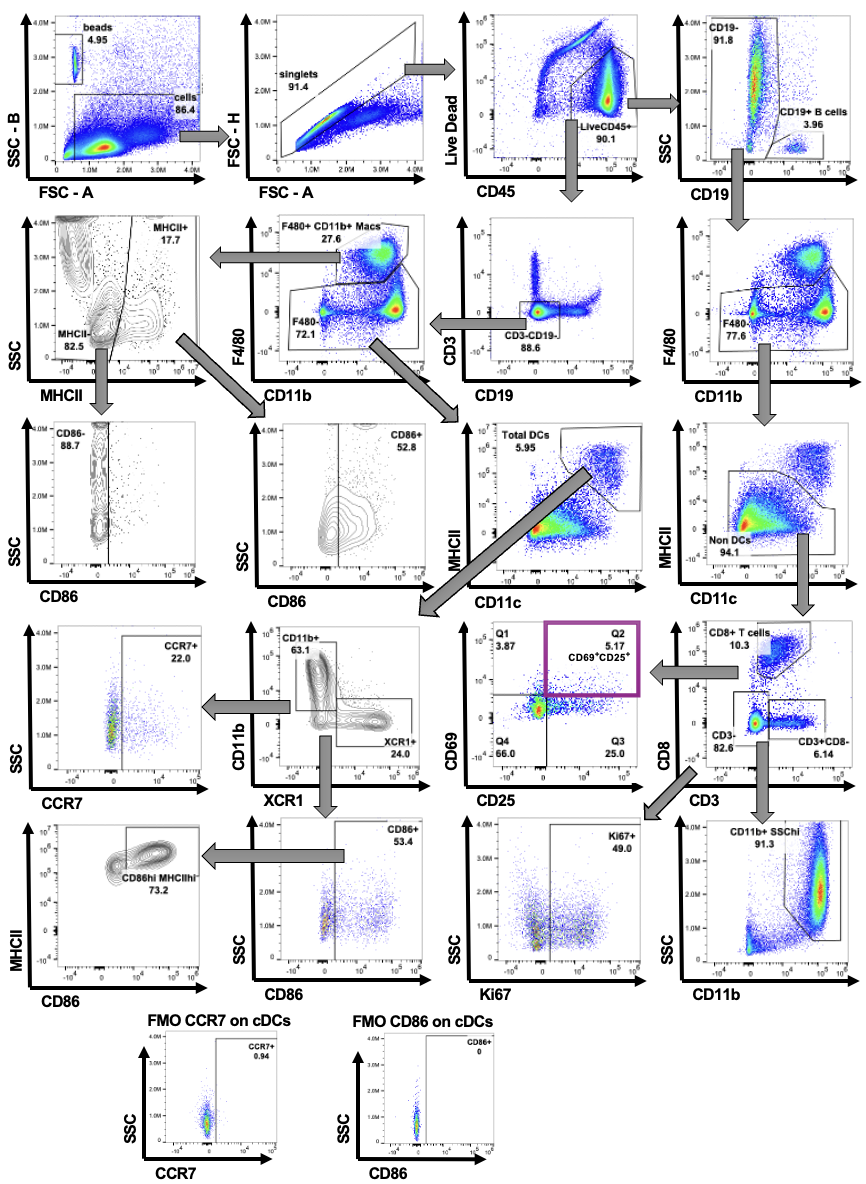


**Supplemental Figure 2. Gating strategy for B16-ZsG tumor flow cytometry analysis in Figures 2-7 and Figures S3, S9-10, S16, S19, S22 & S23.** Gating strategy presented for the following immune cell subsets: F4/80^+^ Macrophages (Live/CD45^+^CD3^-^CD19^-^CD11b^+^F4/80^+^); Conventional Dendritic Cells (cDCs; Live/CD45^+^CD3^-^CD19^-^F4/80^-^CD11c^+^MHCII^+^); cDC1s (Live/CD45^+^CD3^-^CD19^-^F4/80^-^CD11c^+^MHCII^+^XCR1^+^); cDC2s (Live/CD45^+^CD3^-^CD19^-^F4/80^-^CD11c^+^MHCII^+^ CD11b^+^); B-cells (Live/CD45^+^CD19^+^); CD8^+^ T-cells (Live/CD45^+^CD19^-^F4/80^-^Non DCs CD3^+^CD8^+^); CD8^-^ T-cells (Live/CD45^+^CD19^-^F4/80^-^Non DCs CD3^+^CD8^-^); Granulocytes (Live/CD45^+^CD19^-^F4/80^-^Non DCs CD3^-^CD11b^+^SSC^hi^). All frequencies shown are of the parent gate.


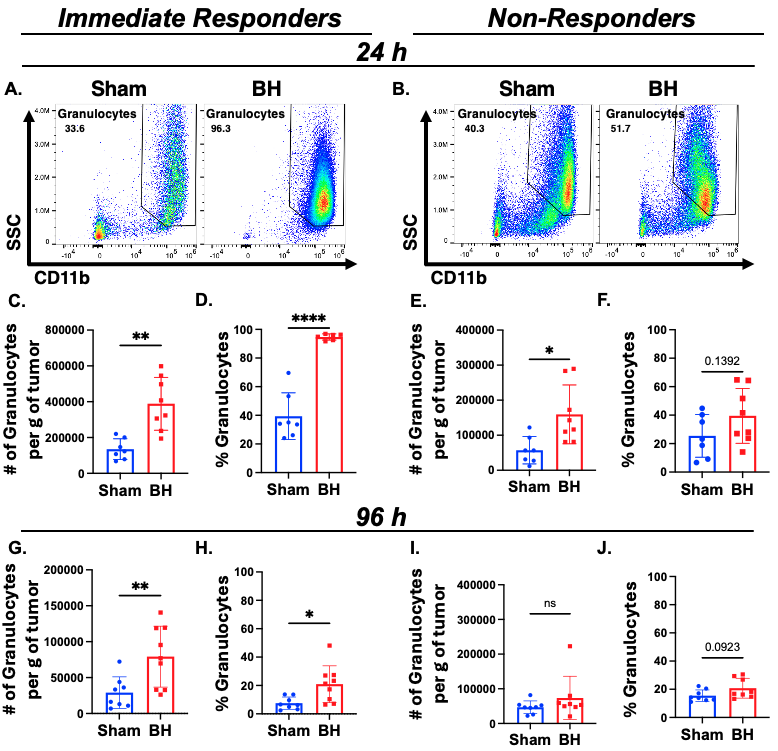


**Supplemental Figure 3. In the absence of BH-induced tumor growth control response, granulocyte presence is still increased in the TME of BH-treated B16-ZsG melanoma tumors. A&B.** Representative flow plots of granulocyte [DUMP(CD3/CD19/Ly6G)^+^CD11b^+^SSC^hi^] presence in the TME of immediate responders (**A**) and non-responders (**B**) 24 h post BH. Frequencies shown are of Live/CD45^+^ population. **C&E**. Number of granulocytes per g of tumor 24 h post treatment in immediate responders (**C**) and non-responders (**E**). **D&F**. Proportion of Live/CD45^+^ cells in the TME that are granulocytes 24 h post treatment in immediate responders (**D**) and non-responders (**F**). **G&I**. Number of granulocytes per g of tumor 96 h post treatment in immediate responders (**G**) and non-responders (**I**). **H&J**. Proportion of Live/CD45^+^ cells in the TME that are granulocytes 96 h post treatment in immediate responders (**H**) and non-responders (**J**). (n=7-8) Unpaired t test with Welch’s correction: ∗ P<0.05, ∗∗ P<0.01, ∗∗∗∗ P<0.0001; Mean ± SD**.** ROUT Outliers analysis with Q=0.1%.


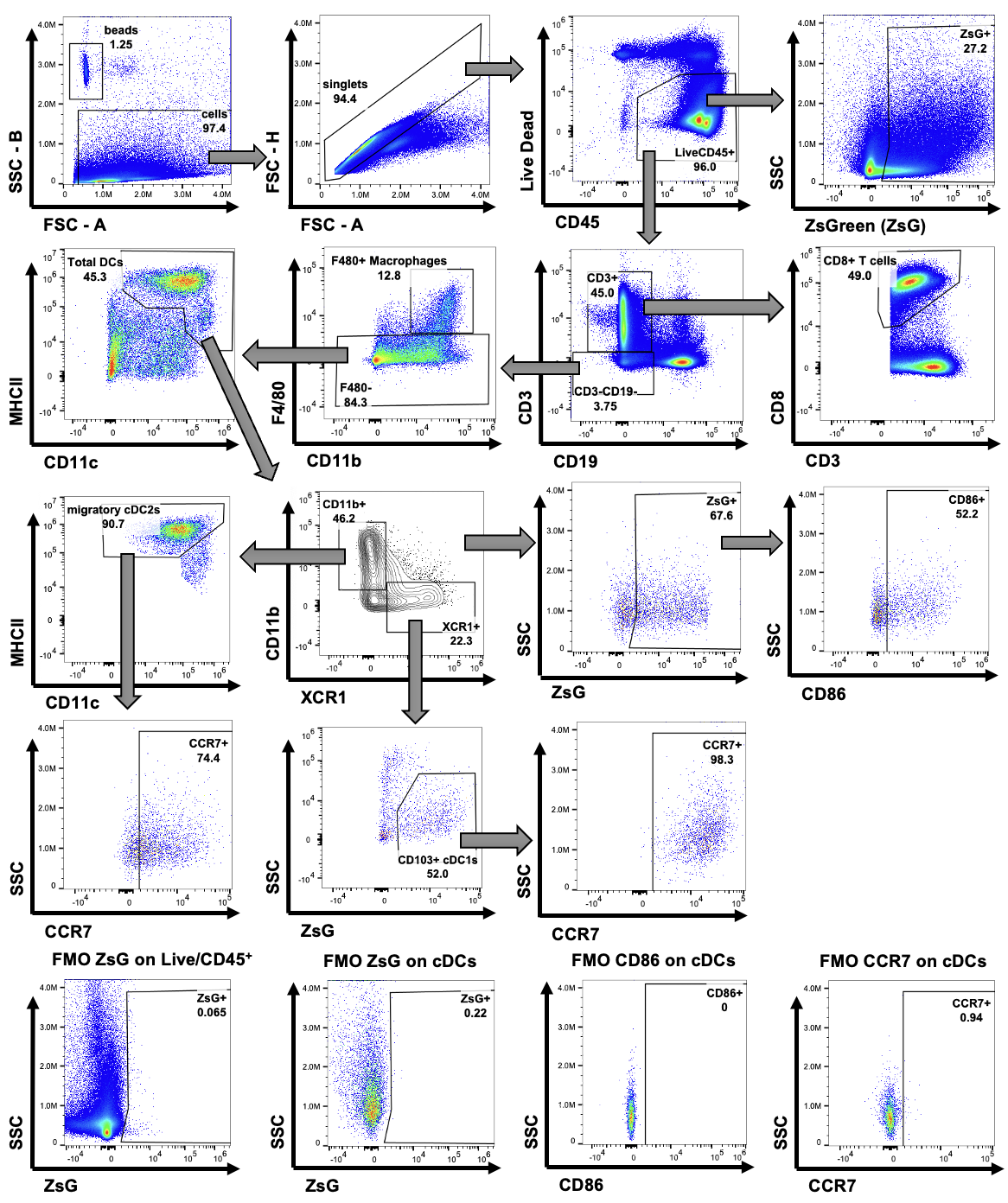


**Supplemental Figure 4. Gating strategy for TDLN flow cytometry analysis in Figures S5, S11, S15 & Figure 8.** Gating strategy presented for the following immune cell subsets: CD8^+^ T-cells (Live/CD45^+^CD3^+^CD8 ^+^); F4/80^+^ Macrophages (Live/CD45^+^CD3^-^CD19^-^F4/80^+^CD11b^+^); Conventional Dendritic Cells (cDCs; Live/CD45^+^CD3^-^CD19^-^F4/80^-^CD11c^+^MHCII^+^); cDC1s (Live/CD45^+^CD3^-^CD19^-^F4/80^-^CD11c^+^MHCII^+^ XCR1^+^); migratory cDC1s (Live/CD45^+^CD3^-^CD19^-^F4/80^-^CD11c^+^MHCII^+^ XCR1^+^ CD103^+^); cDC2s (Live/CD45^+^CD3^-^CD19^-^F4/80^-^CD11c^+^MHCII^+^CD11b^+^); migratory cDC2s (Live/CD45^+^CD3^-^CD19^-^F4/80^-^CD11c^+^MHCII^+^CD11b^+^MHCII^hi^). All frequencies shown are of the parent gate.


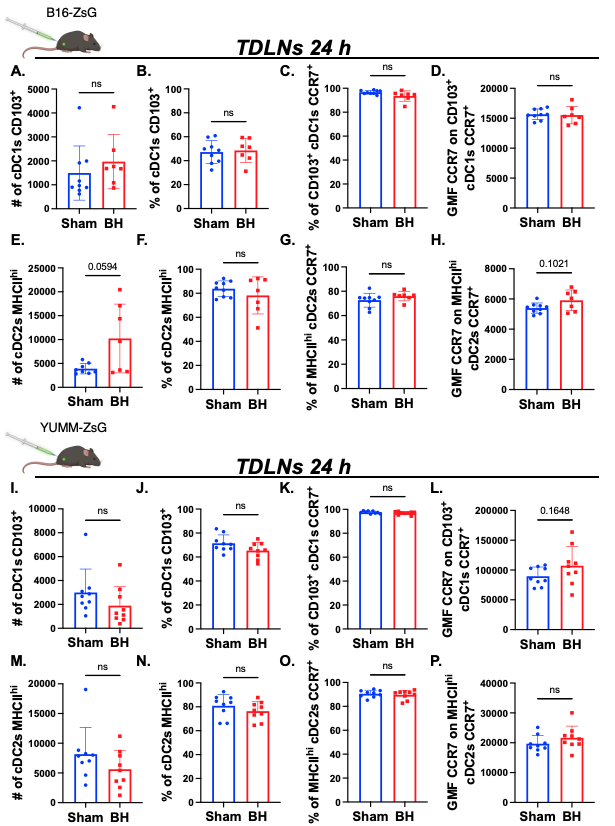


**Supplemental Figure 5. Immediate growth control in response to BH did not result in significant changes to migratory cDCs in the TDLNs 24 h post treatment. A&I.** The number of migratory cDC1s in B16-ZsG (**A**) or YUMM-ZsG (**I**) TDLNs. **B&J.** The proportion of cDC1s that are migratory in B16-ZsG (**B**) or YUMM-ZsG (**J**) TDLNs. **C&K.** The proportion of migratory cDC1s that are CCR7^+^ in B16-ZsG (**C**) or YUMM-ZsG (**K**) TDLNs. **D&L.** The GMF of CCR7 on migratory cDC1s in B16-ZsG (**D**) or YUMM-ZsG (**L**) TDLNs. **E&M.** The number of migratory cDC2s in B16-ZsG (**E**) or YUMM-ZsG (**M**) TDLNs. **F&N.** The proportion of cDC2s that are migratory in B16-ZsG (**F**) or YUMM-ZsG (**N**) TDLNs. **G&O.** The proportion of migratory cDC2s that are CCR7^+^ in B16-ZsG (**G**) or YUMM-ZsG (**O**) TDLNs. **H&P.** The GMF of CCR7 on migratory cDC2s in B16-ZsG (**H**) or YUMM-ZsG (**P**) TDLNs. (n=7-9) Unpaired t test with Welch’s correction: ns = nonsignificant; Mean ± SD**.** ROUT Outliers analysis with Q=0.1%.


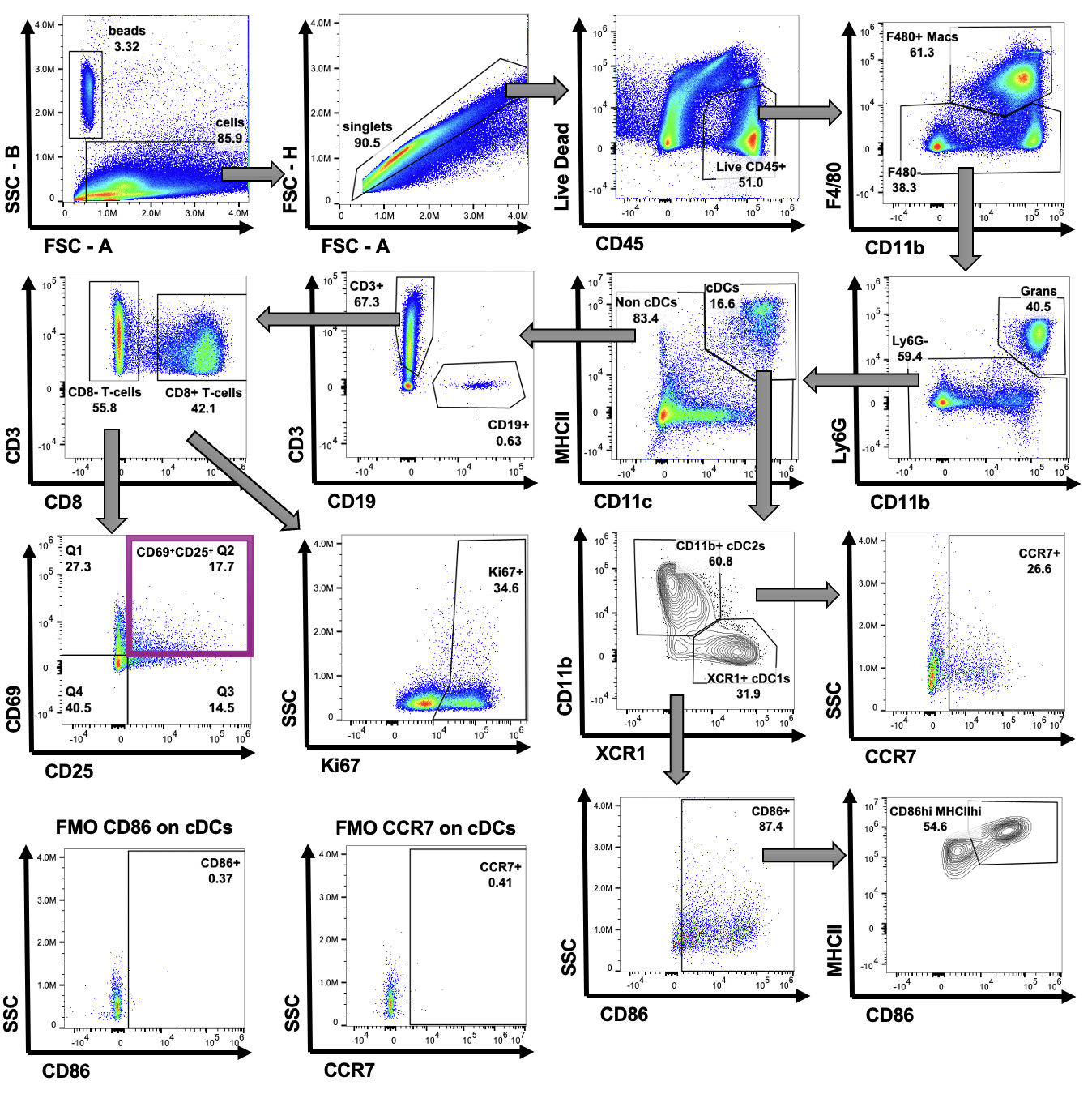


**Supplemental Figure 6. Gating strategy for YUMM-ZsG tumor flow cytometry analysis in Figures S7-8, S10, S14-15, S17-18, S20 & S23.** Gating strategy presented for the following immune cell subsets: Macrophages (Live/CD45^+^CD11b^+^F4/80^+^); Granulocytes (Live/CD45^+^F4/80^-^Ly-6G^+^CD11b^+^); Conventional Dendritic Cells (cDCs; Live/CD45^+^F4/80^-^Ly-6G^-^CD11c^+^MHCII^+^); cDC1s (Live/CD45^+^F4/80^-^Ly-6G^-^CD11c^+^MHCII^+^XCR1^+^); cDC2s (Live/CD45^+^F4/80^-^Ly-6G^-^CD11c^+^MHCII^+^CD11b^+^); B-cells (Live/CD45^+^F4/80^-^Ly-6G^-^Non DCs CD19^+^); CD8^+^ T-cells (Live/CD45^+^F4/80^-^Ly-6G^-^Non DCs CD3^+^CD8^+^); CD8^-^ T-cells (Live/CD45^+^F4/80^-^Ly-6G^-^Non DCs CD3^+^CD8^-^). All frequencies shown are of the parent gate.


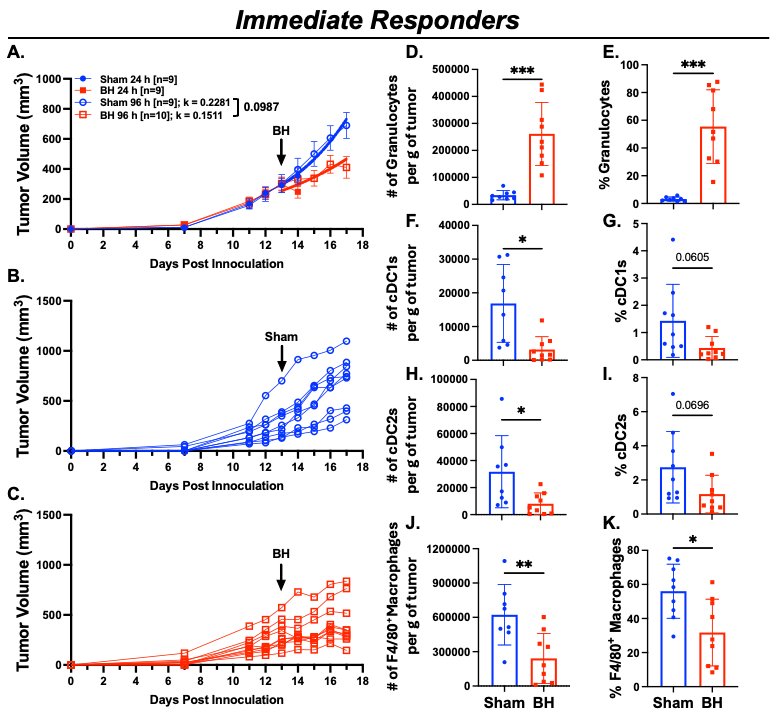


**Supplemental Figure 7. BH-induced immediate growth control is associated with reduced APC presence in the YUMM-ZsG TME 24 h post ablation. A.** Average tumor growth curves for YUMM-ZsG tumor-bearing mice treated with Sham or 1mm-spaced BH on day 13. **B&C.** Individual tumor growth curves of Sham (**B**) or BH (**C**) treated mice averaged in (**A**). **D&E**. Number (**D**) and proportion (**E**) of granulocytes in the TME. **F&G**. Number (**F**) and proportion (**G**) of cDC1s in the TME. **H&I**. Number (**H**) and proportion (**I**) of cDC2s in the TME. **J&K**. Number (**J**) and proportion (**K**) of F4/80^+^ macrophages in the TME. Nonlinear regression model of exponential growth equation on tumor outgrowth from D13-D17 for 96 h cohorts. Mixed-effects model on day 17. All points represent mean ± SEM (**A**). (n=9) Unpaired t test with Welch’s correction: ns = nonsignificant, ∗ P<0.05, ∗∗ P<0.01, ∗∗∗ P<0.001; Mean ± SD**.** ROUT Outliers analysis with Q=0.1% (**D-K**).


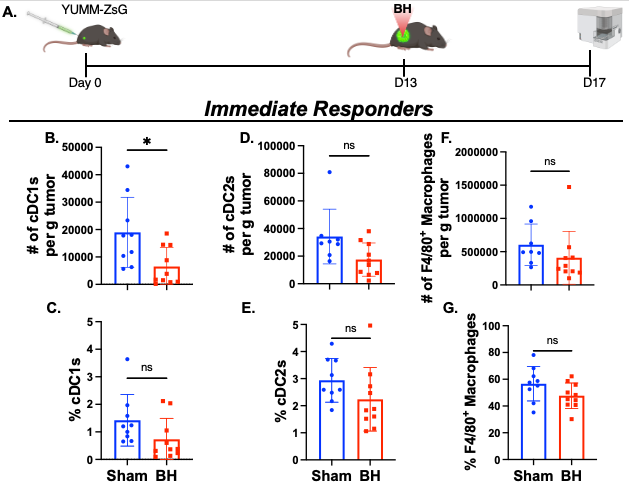


**Supplemental Figure 8. Minimal reduction in APC presence is observed in the TME 96 h post ablation in the YUMM-ZsG model. A**. Schematic for experimental timeline from tumor initiation to BH or Sham treatment followed by 96 h flow cytometry analysis of the tumors. **B&C**. Number (**B**) and proportion (**C**) of cDC1s in the TME. **D&E**. Number (**D**) and proportion (**E**) of cDC2s in the TME. **F&G**. Number (**F**) and proportion (**G**) of F4/80^+^ macrophages in the TME. (n=7-8) Unpaired t test with Welch’s correction: ns = nonsignificant; Mean ± SD**.** ROUT Outliers analysis with Q=0.1%.

*
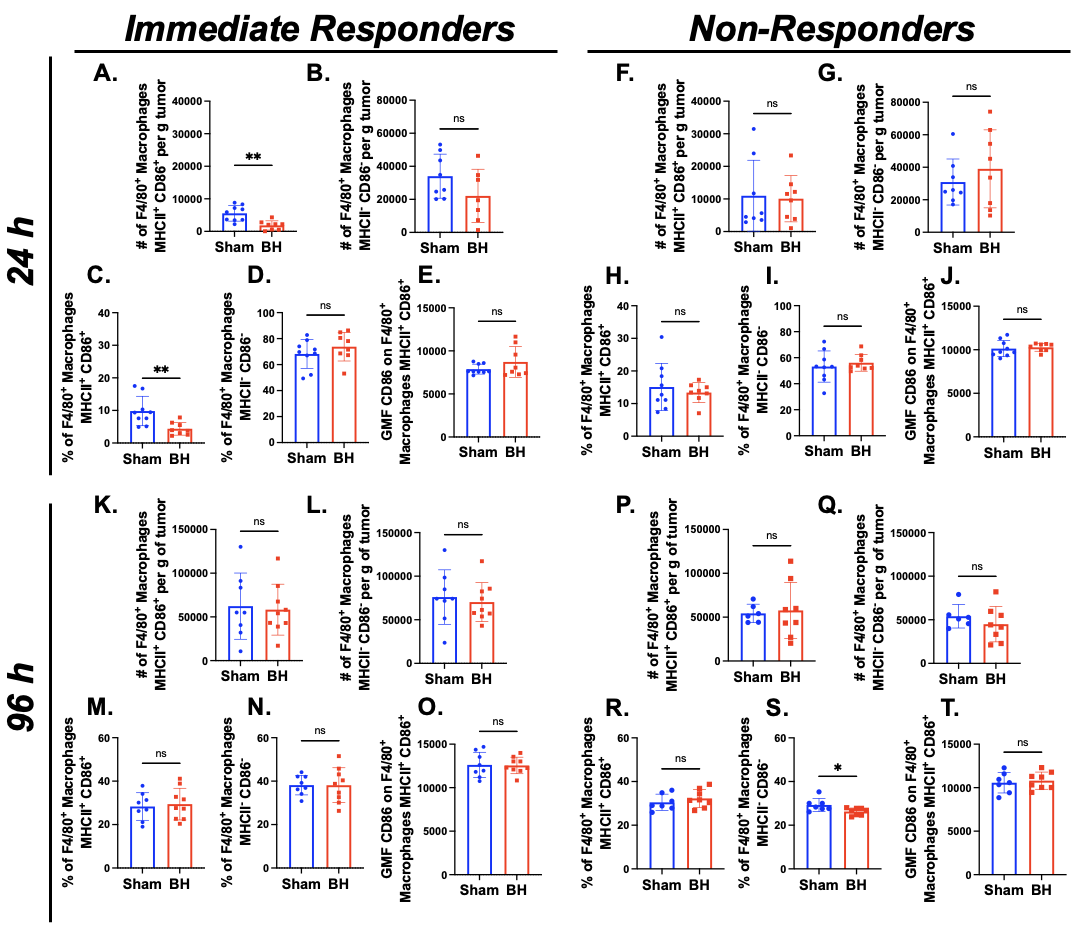
*

**Supplemental Figure 9. Acute growth response to BH does not correspond to a reduced pro-tumorigenic macrophage population in the TME. A-B&K-L**. Number of anti-tumorigenic [MHCII^+^CD86^+^] (**A&K**) or pro-tumorigenic [MHCII^-^CD86^-^] (**B&L**) macrophages in the B16-ZsG TME of immediate responders 24 h or 96 h post treatment, respectively. **C-D&M-N.** Proportion of F4/80^+^ macrophages that are anti-tumorigenic (**C&M**) or pro-tumorigenic (**D&N**) in the TME of immediate responders 24 h or 96 h post treatment, respectively. **E&O.** CD86 intensity on anti-tumorigenic macrophages in the TME of immediate responders 24 h (**E**) or 96 h (**O**) post treatment. **F-G&P-Q**. Number of anti-tumorigenic (**F&P**) or pro-tumorigenic (**G&Q**) macrophages in the B16-ZsG TME of non-responders 24 h or 96 h post treatment, respectively. **H-I&R-S.** Proportion of F4/80^+^ macrophages that are anti-tumorigenic (**H&R**) or pro-tumorigenic (**I&S**) in the TME of non-responders 24 h or 96 h post treatment, respectively. **J&T.** CD86 intensity on anti-tumorigenic macrophages in the TME of non-responders 24 h (**J**) or 96 h (**T**) post treatment. (n=8-9) Unpaired t test with Welch’s correction: ns = nonsignificant, ∗ P<0.05, ∗∗ P<0.01; Mean ± SD**.** ROUT Outliers analysis with Q=0.1%.


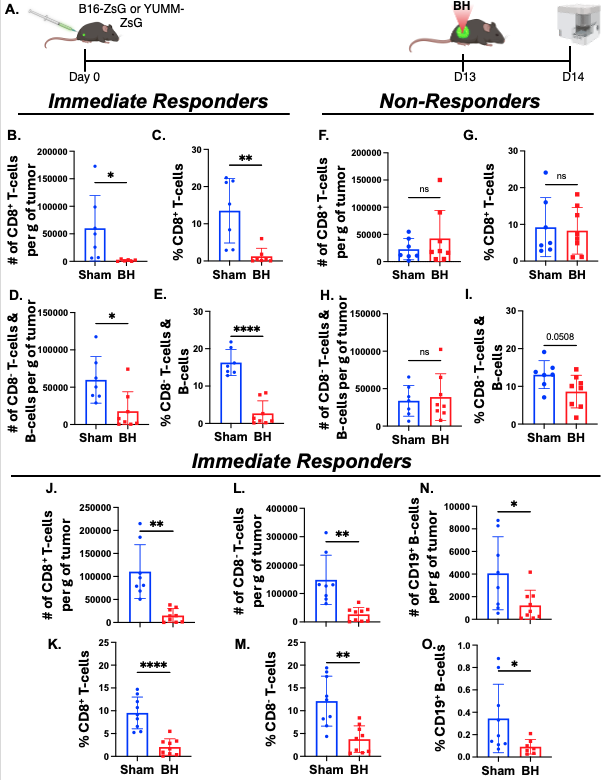


**Supplemental Figure 10. BH-induced immediate growth control is associated with reduced lymphocyte presence in the TME. A**. Schematic for experimental timeline from tumor initiation to BH or Sham treatment followed by 24 h flow cytometry analysis of the tumors. **B&F**. Number of CD8^+^ T-cells [DUMP(CD3/CD19/Ly6G)^+^CD8^+^] per g of B16-ZsG tumor in the immediate responders (**B**) and non-responders (**F**). **C&G**. Proportion of Live/CD45^+^ cells in the B16-ZsG TME that are CD8^+^ T-cells in immediate responders (**C**) and non-responders (**G**). **D&H**. Number of CD8^-^ T-cells & B-cells [DUMP(CD3/CD19/Ly6G)^+^CD8^-^CD11b^-^SSC^lo^] per g of B16-ZsG tumor in the immediate responders (**D**) and non-responders (**H**). **E&I**. Proportion of Live/CD45^+^ cells in the B16-ZsG TME that are CD8^-^ T-cells & B-cells in immediate responders (**E**) and non-responders (**I**). **J**. Number of CD8^+^ T-cells per g of YUMM-ZsG tumor in the immediate responders. **K**. Proportion of Live/CD45^+^ cells in the YUMM-ZsG TME that are CD8^+^ T-cells. **L**. Number of CD8^-^ T-cells per g of YUMM-ZsG tumor in the immediate responders. **M**. Proportion of Live/CD45^+^ cells in the YUMM-ZsG TME that are CD8^-^ T-cells. **N**. Number of CD19^+^ B-cells per g of YUMM-ZsG tumor in the immediate responders. **O**. Proportion of Live/CD45^+^ cells in the YUMM-ZsG TME that are CD19^+^ B-cells. (n=7-9) Unpaired t test with Welch’s correction: ns = nonsignificant, ∗ P<0.05, ∗∗ P<0.01, ∗∗∗∗ P<0.0001; Mean ± SD**.** ROUT Outliers analysis with Q=0.1%.

*
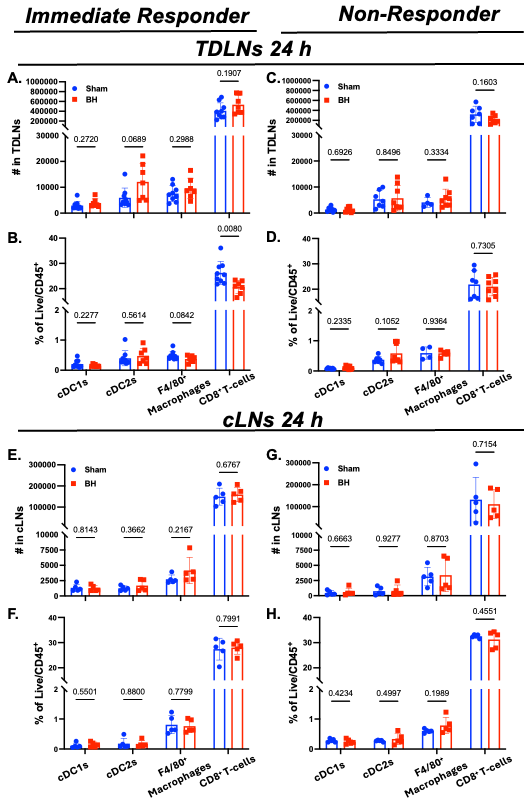
*

**Supplemental Figure 11. APC and CD8^+^ T-cell presence in TDLNs and cLNs 24 h post treatment. A&B.** The number (**A**) and the proportion (**B**) of APCs and CD8^+^ T-cells in the TDLNs of immediate responders. **C&D.** The number (**C**) and the proportion (**D**) of APCs and CD8^+^ T-cells in the TDLNs of non-responders. (n=7-9) **E&F.** The number (**E**) and the proportion (**F**) of APCs and CD8^+^ T-cells in the cLNs of immediate responders. **G&H.** The number (**G**) and the proportion (**H**) of APCs and CD8^+^ T-cells in the TDLNs of non-responders. (n=5) Multiple unpaired t test with Welch’s correction; Mean ± SD**.** ROUT Outliers analysis with Q=0.1%.


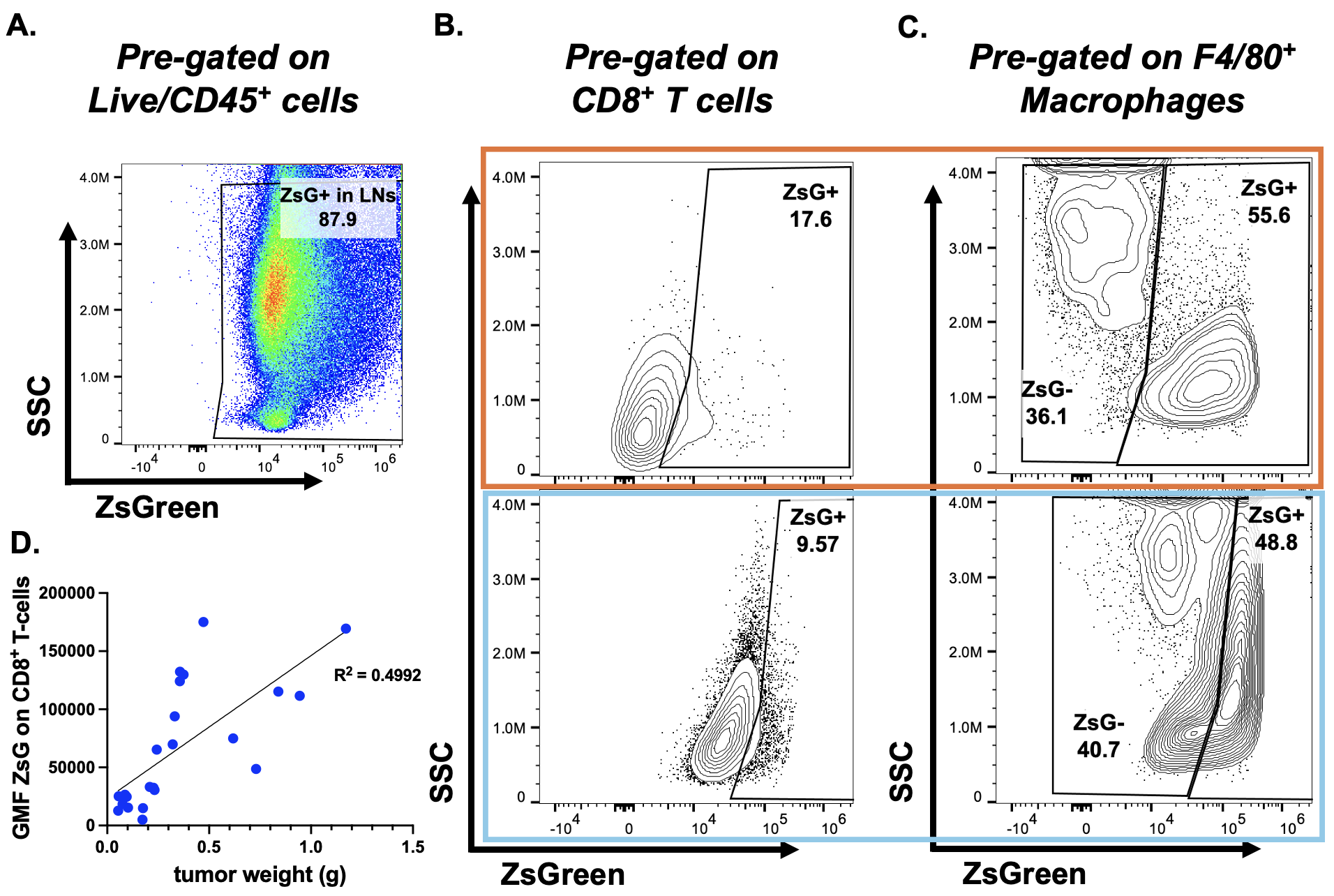


**Supplemental Figure 12. Identifying ZsGreen in the TME requires individualized gating on CD8^+^ T-cells as a biologically negative control. A.** Representative flow plot of the ZsG gate used in the TDLNs based on a ZsGreen naïve non-tumor-bearing control on Live/CD45^+^ cells in the TME. **B.** Representative flow plots of CD8^+^ T-cells being used to set up the ZsG^+^ gate for each sample. **C.** Representative scatter plots showing ZsG expression in F4/80^+^ macrophages. The orange and blue boxes represent matched samples from the same tumor. **D.** Linear regression correlation between the GMF of ZsG on CD8^+^ T-cells and tumor weight (g). Simple linear regression model.


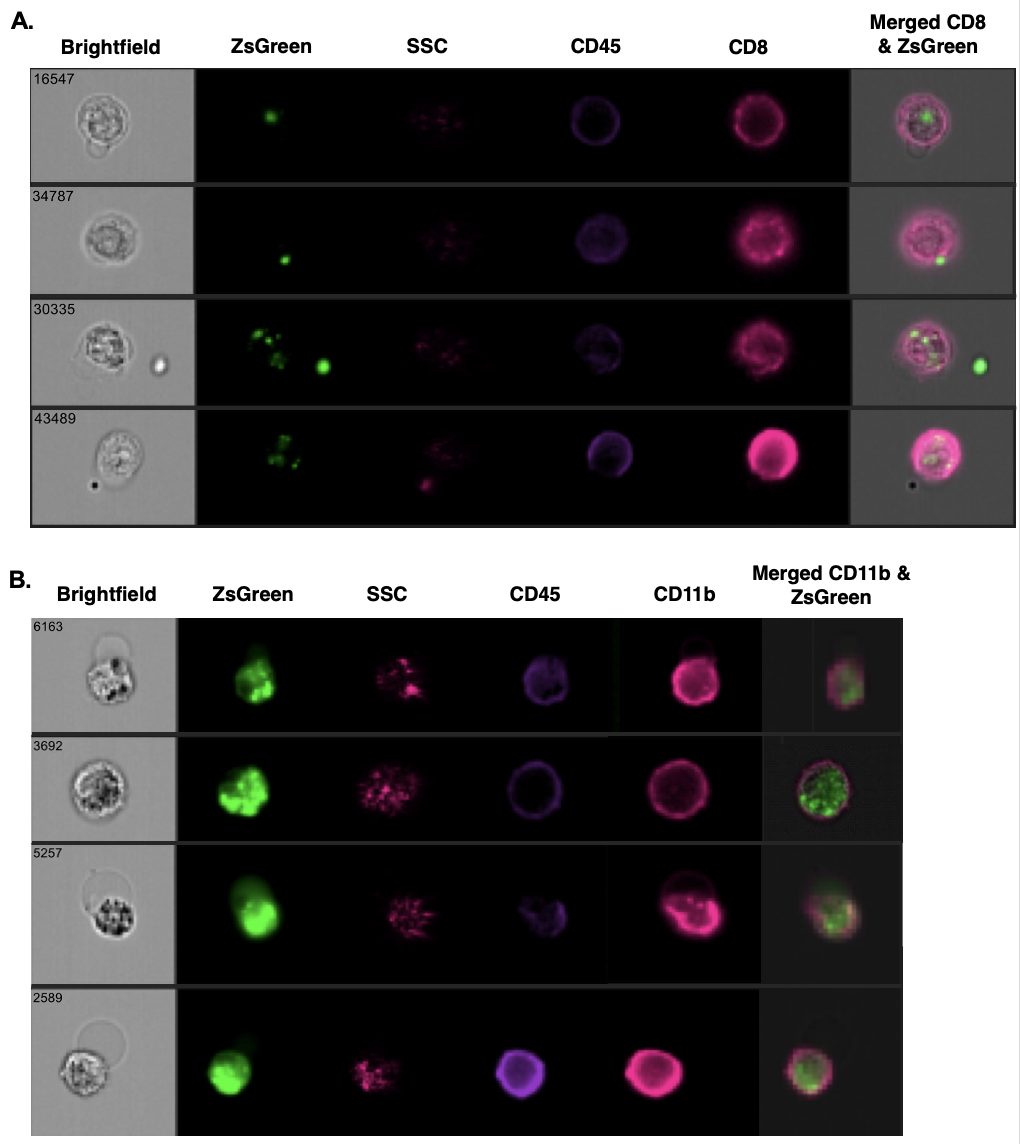


**Supplemental Figure 13. ImageStream microscopy showing distinct ZsGreen expression on CD8^+^ and CD11b^+^ immune cells in the TME. A.** Representative images of CD45^+^CD8^+^ cells from B16-ZsG tumors expressing punctate ZsGreen. Pre-gated on live cells. **B.** Representative images of CD45^+^CD11b^+^ cells from B16-ZsG tumors expressing diffuse ZsGreen. Pre-gated on live cells.


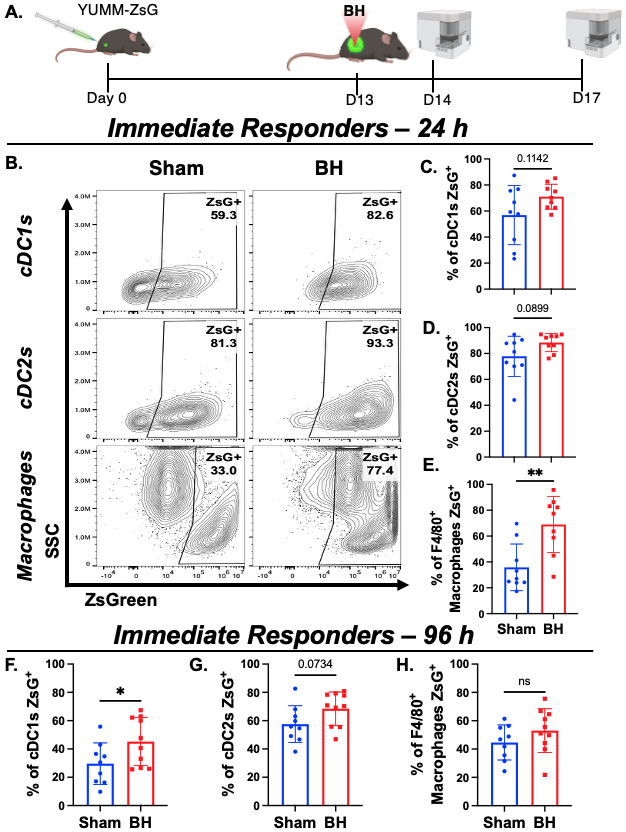


**Supplemental Figure 14. Increased** **tumor antigen acquisition is also observed when BH-induces constraint of YUMM-ZsG melanoma tumors. A**. Schematic for experimental timeline from tumor initiation to BH or Sham treatment followed by 24 h and 96 h flow cytometry analysis of the tumors. **B.** Representative flow plots where an immediate growth control response was observed post BH, showing ZsG acquisition in cDC1s (top), cDC2s (middle), and F4/80^+^ macrophages (bottom) in the TME of Sham and BH cohorts 24 h post treatment. Frequencies shown are of the parent population. **C-E.** Proportion of cDC1s (**C**), cDC2s (**D**), and F4/80^+^ macrophages (**E**) that are ZsG^+^ 24 h post treatment. **F-H.** Proportion of cDC1s (**F**), cDC2s (**G**), and F4/80^+^ macrophages (**H**) that that are ZsG^+^ 96 h post treatment. (n=9-10) Unpaired t test with Welch’s correction: ns = nonsignificant, ∗ P<0.05, ∗∗ P<0.01; Mean ± SD**.** ROUT Outliers analysis with Q=0.1%


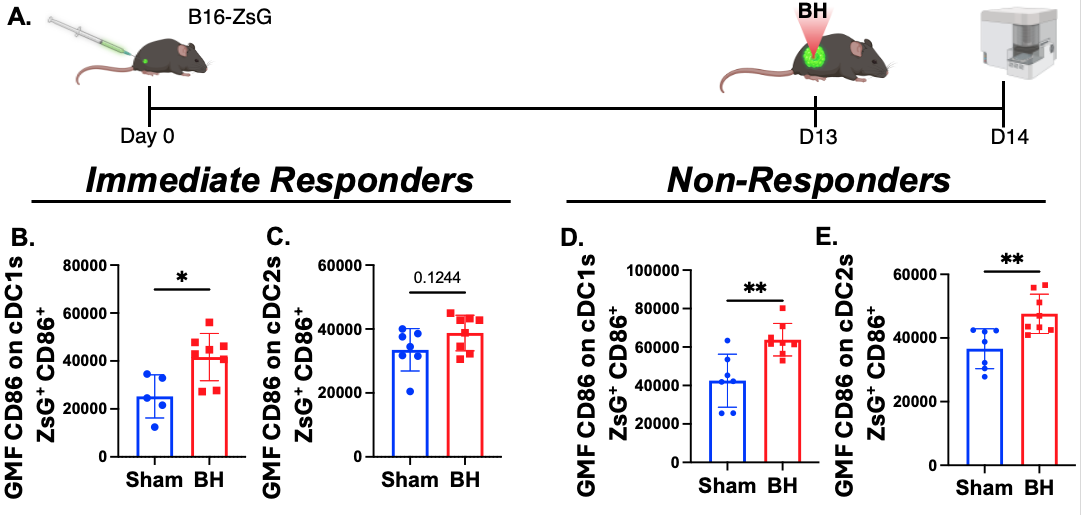


**Supplemental Figure 15. BH treatment induces cDC phenotypic activation in an antigen dependent manner in the TDLNs 24 h post treatment independent of tumor growth control response. A**. Schematic for experimental timeline from tumor initiation to BH or Sham treatment followed by 24 h flow cytometry analysis of the TDLNs. **B&D.** The intensity of CD86 on ZsG^+^ CD86^+^ cDC1s in the TDLNs post treatment in the immediate responders (**B**) and the non-responders (**D**). **C&E.** The intensity of CD86 on ZsG^+^ CD86^+^ cDC2s in the TDLNs post treatment in the immediate responders (**C**) and the non-responders (**E**). (n=7-8) Unpaired t test with Welch’s correction: ∗ P<0.05, ∗∗ P<0.01; Mean ± SD**.** ROUT Outliers analysis with Q=0.1%.


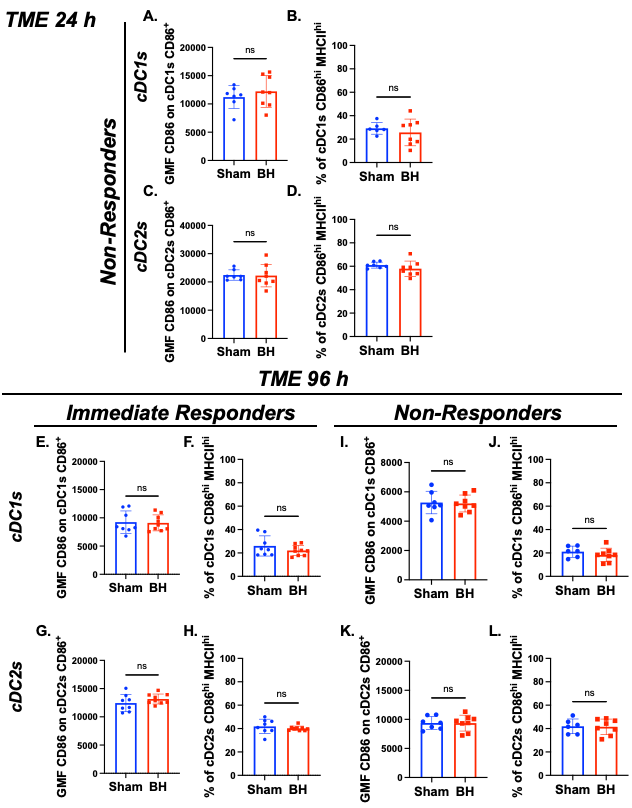


**Supplemental Figure 16. Tumor response to BH is associated with acute elevated activation of cDCs in the B16-ZsG melanoma model. A&I.** The intensity of CD86 on total cDC1s CD86^+^ in non-responsive tumors 24 h (**A**) and 96 h (**I**) post BH. **B&J.** Proportion of cDC1s phenotypically activated (CD86^hi^MHCII^hi^) in the TME of non-responders 24 h (**B**) and 96 h (**J**) post BH. **C&K.** The intensity of CD86 on total cDC2s CD86^+^ in non-responsive tumors 24 h (**C**) and 96 h (**K**) post BH. **D&L.** Proportion of cDC2s phenotypically activated in the TME of non-responders 24 h (**D**) and 96 h (**L**) post BH. **E.** The intensity of CD86 on total cDC1s CD86^+^ in the responsive tumors 96 h post BH. **F.** Proportion of cDC1s phenotypically activated in the TME of responders 96 h post BH. **G.** The intensity of CD86 on total cDC2s CD86^+^ in responsive tumors 96 h post BH. **H.** Proportion of cDC2s phenotypically activated in the TME of responders 96 h post BH. (n=8-9) Unpaired t test with Welch’s correction: ns = nonsignificant, ∗∗ P<0.01, ∗∗∗ P<0.001; Mean ± SD**.** ROUT Outliers analysis with Q=0.1%.


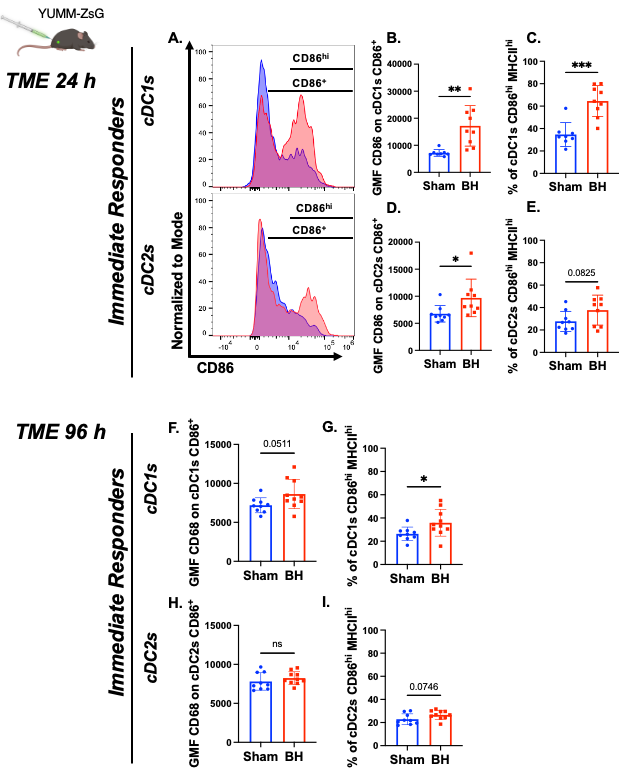


**Supplemental Figure 17. Tumor constraint by BH is associated with elevated phenotypic activation of cDCs in the YUMM-ZsG melanoma model. A**. Histograms showing CD86 intensity on cDC1s (top) and cDC2s (bottom) after treatment with BH or Sham controls in immediate responders. **B&F.** The intensity of CD86 on cDC1s CD86^+^ in the TME 24 h (**B**) and 96 h (**F**) post BH. **C&G.** Proportion of cDC1s phenotypically activated (CD86^hi^MHCII^hi^) in the TME 24 h (**C**) and 96 h (**G**) post BH. **D&H.** The intensity of CD86 on cDC2s CD86^+^ in the TME 24 h (**D**) and 96 h (**H**) post BH. **E&I.** Proportion of cDC2s phenotypically activated in the TME 24 h (**E**) and 96 h (**I**) post BH. (n=9-10) Unpaired t test with Welch’s correction: ∗ P<0.05, ∗∗ P<0.01, ∗∗∗ P<0.001; Mean ± SD**.** ROUT Outliers analysis with Q=0.1%.


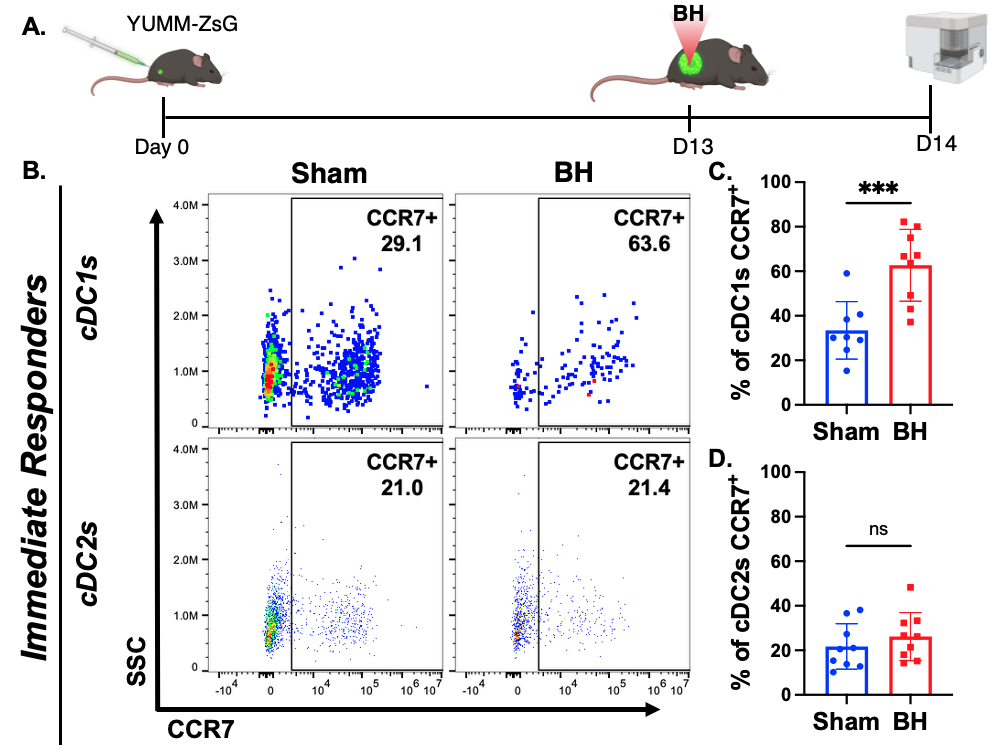


**Supplemental Figure 18. BH-induced tumor constrain is associated with an increased frequency of CCR7-expressing cDC1s in the TME of the YUMM-ZsG model. A**. Schematic for experimental timeline from tumor initiation to BH or Sham treatment followed by 24 h flow cytometry analysis of the tumors. **B.** Scatter plots showing CCR7 frequency on cDC1s (top) and cDC2s (bottom) after treatment with BH or Sham controls in immediate responders. **C.** Proportion of cDC1s that are CCR7^+^ in the TME. **D.** Proportion of cDC2s that are CCR7^+^ in the TME. (n=9-10) Unpaired t test with Welch’s correction: ns = nonsignificant, ∗∗∗ P<0.001; Mean ± SD**.** ROUT Outliers analysis with Q=0.1%.


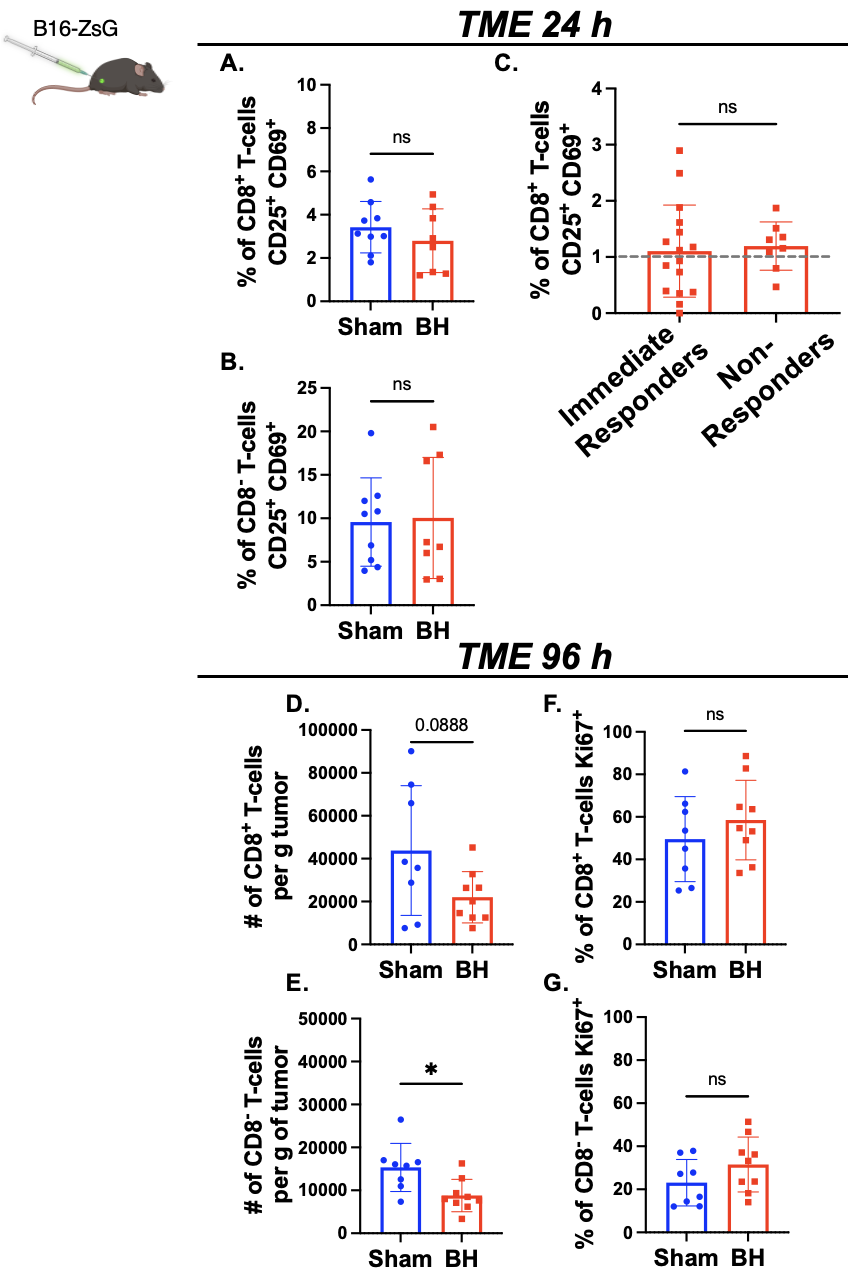


**Supplemental Figure 19. Immediate growth control in response to BH in the B16-ZsG model is not associated with broad T-cell activation or expansion in the TME. A&B**. Proportion of CD8^+^ (**A**) or CD8^-^ (**B**) T-cells in the TME that are phenotypically activated [CD69^+^CD25^+^] 24 h post treatment. **C.** Fold change for the proportion of CD8^+^ T-cells that are CD69^+^CD25^+^ in immediate responders and non-responders normalized to the average of their respective Sham control cohort. **D&E**. Number of CD8^+^ (**D**) or CD8^-^ (**E**) T-cells per g of tumor 96 h post treatment. **F&G.** Proportion of CD8^+^ (**F**) or CD8^-^ (**G**) T-cells in the B16-ZsG TME 96 h post treatment that are Ki67^+^. (n=8-9 or n=16 immediate responders & n=8 non-responders) Unpaired t test with Welch’s correction: ns = nonsignificant, ∗ P<0.05; Mean ± SD**.** ROUT Outliers analysis with Q=0.1%.


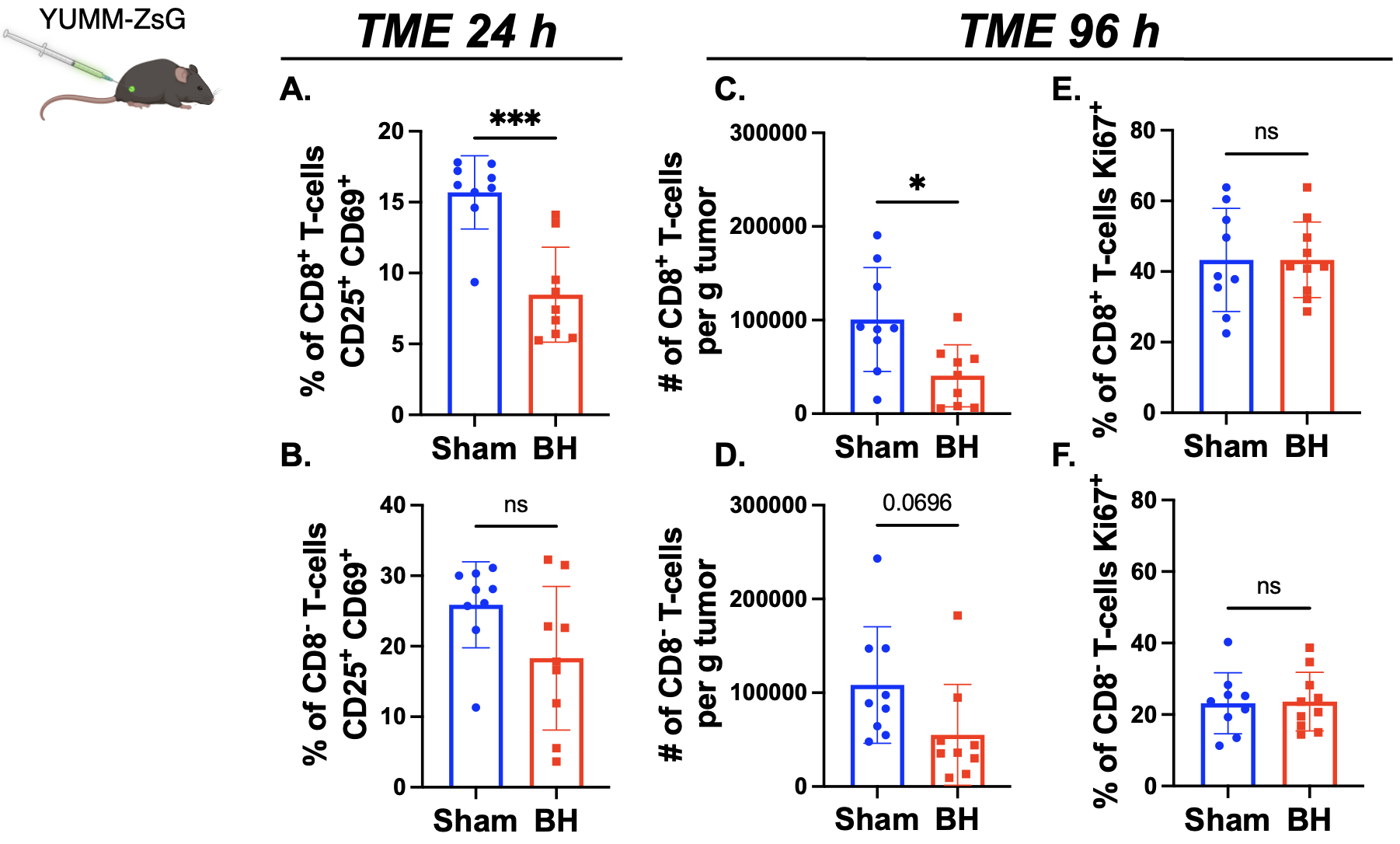


**Supplemental Figure 20. Immediate growth control in response to BH in the YUMM-ZsG model is not associated with broad T-cell activation or expansion in the TME. A&B**. Proportion of CD8^+^ (**A**) or CD8^-^ (**B**) T-cells in the TME that are phenotypically activated [CD69^+^CD25^+^] 24 h post treatment. **C&D**. Number of CD8^+^ (**C**) or CD8^-^ (**D**) T-cells per g of tumor 96 h post treatment. **E&F.** Proportion of CD8^+^ (**E**) or CD8^-^ (**F**) T-cells in the YUMM-ZsG TME 96 h post treatment that are Ki67^+^. (n=8-9) Unpaired t test with Welch’s correction: ns = nonsignificant, ∗ P<0.05, ∗∗∗ P<0.001; Mean ± SD**.** ROUT Outliers analysis with Q=0.1%.


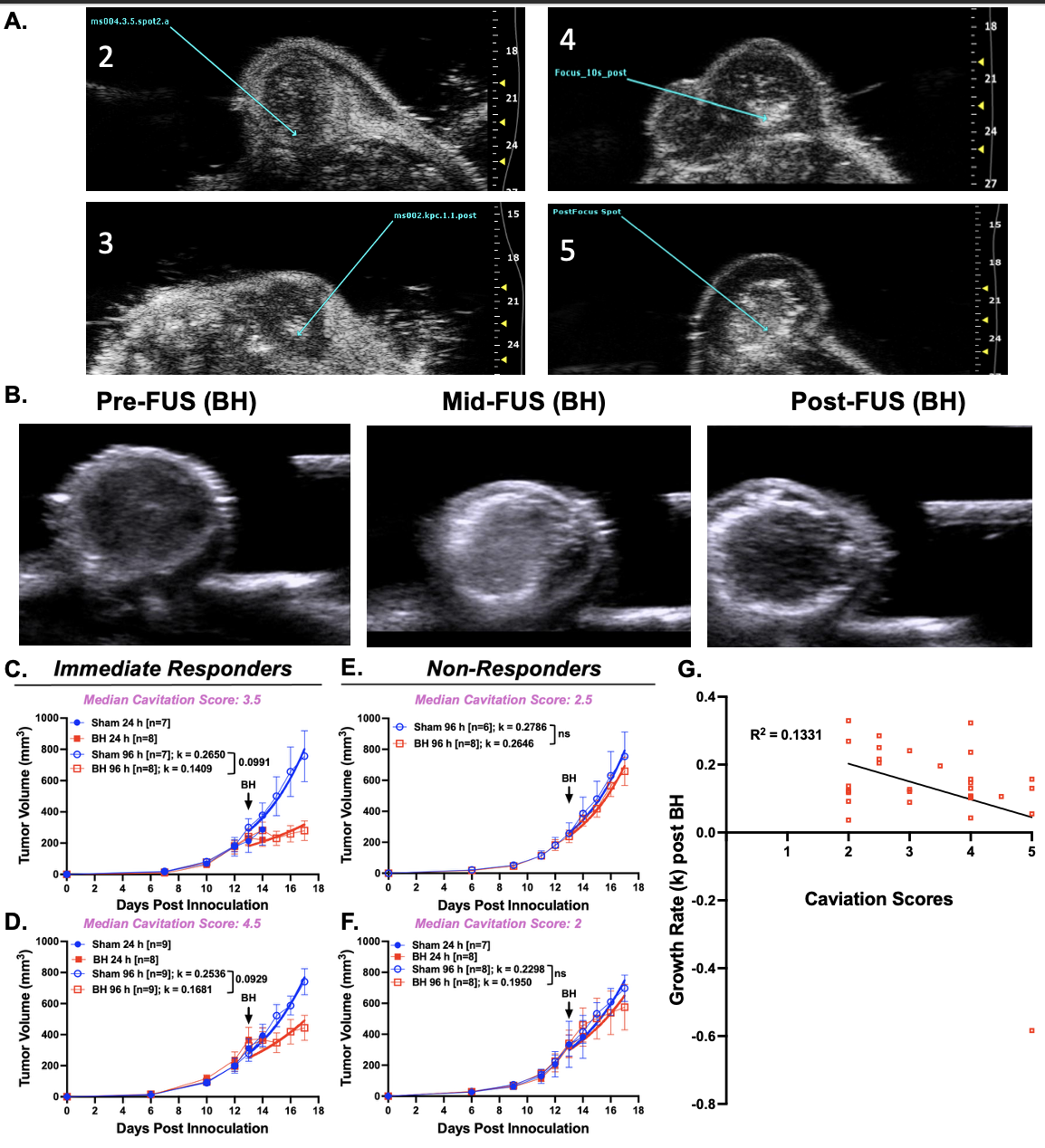


**Supplemental Figure 21. While higher median cavitation scores appear to correspond with the immediate responders, there is not a direct correlation between cavitation score and tumor outgrowth rate post BH. A.** Representative images of the qualitative cavitation scoring system from 2-5 based on real time B-mode imaging occurring during treatment. **B.** Representative images of single B16-ZsG tumor before, during, and after BH treatment for when a Cavitation Score of a 5 was achieved. **C-F.** Average tumor growth curves for B16-ZsG tumor-bearing mice treated with Sham or 1mm-spaced BH on day 13 from four separate experiments. Median cavitation scores for the 96 h BH cohorts displayed for each experiment. **G.** Linear regression correlation between cavitation score and tumor outgrowth rate post BH treatment (n=32). Non-linear regression model on tumor outgrowth from D13-D17 for 96 h cohorts. Mixed-effects model on day 17; ns = nonsignificant. All points represent Mean ± SEM (**C-F**). Simple linear regression model (**G**).

**
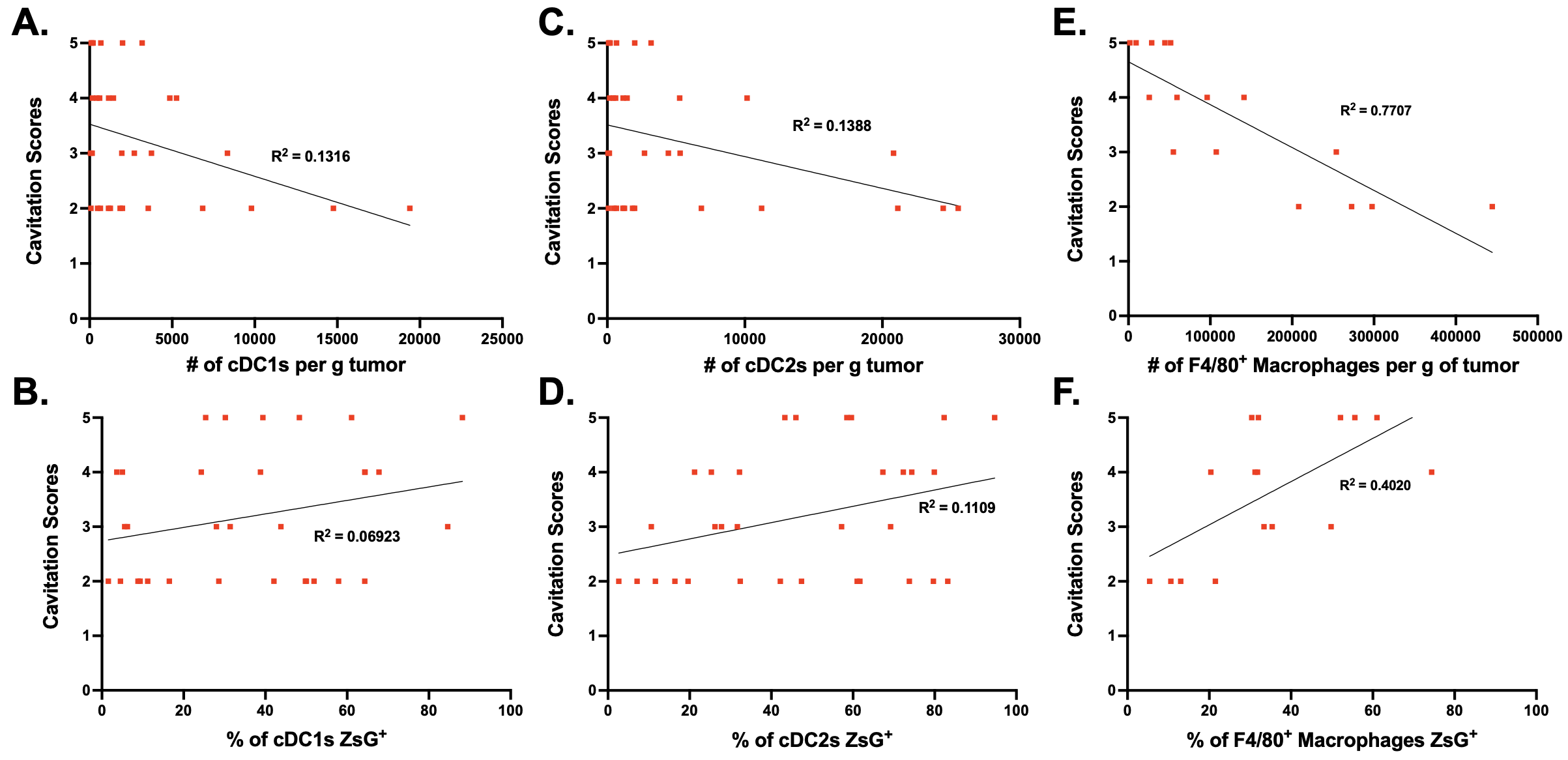
**

**Supplemental Figure 22. Correlations between cavitation scoring and APC presence and tumor antigen acquisition in the TME 24 h post BH. A&B.** Linear regression correlation between cavitation score and the number of cDC1s (**A**) and the proportion ZsG^+^ (**B**) in B16-ZsG tumors 24 h post BH treatment. **C&D.** Linear regression correlation between cavitation score and the number of cDC2s (**C**) and the proportion ZsG^+^ (**D**) in B16-ZsG tumors 24 h post BH treatment. (n=32) **E&F.** Linear regression correlation between cavitation score and the number of F4/80^+^ macrophages (**E**) and the proportion ZsG^+^ (**F**) in B16-ZsG tumors 24 h post BH treatment. (n=16) Simple linear regression model.


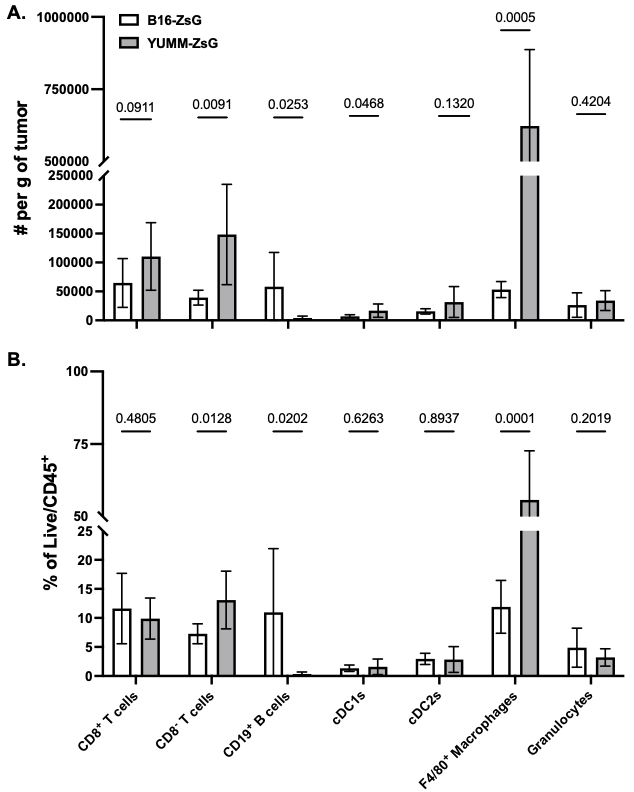


**Supplemental Figure 23. Differences in immune populations present in Sham untreated B16-ZsG and YUMM-ZsG tumors. A.** Number of various immune populations present in B16-ZsG (white) versus YUMM-ZsG (gray) tumors. **B.** Proportions of various immune populations of total Live/CD45^+^ immune cells in B16-ZsG (white) versus YUMM-ZsG (gray) tumors. (n=8-9) Multiple unpaired t test with Welch’s correction; Mean ± SD**.** ROUT Outliers analysis with Q=0.1%.

**Table S1. Key Resources Table**

| REAGENTS | SOURCE | IDENTIFIER |
| --- | --- | --- |
| Anti-mouse CD45 clone 30-F11 BUV395 | BD | Cat# 564279; RRID: AB_2651134 |
| Anti-mouse CD19 clone 1D3 BUV737 | BD | Cat# 612781; RRID: AB_2870111 |
| Anti-mouse MHCII clone M5/114 BUV496 | BD | Cat# 750281; RRID: AB_2874472 |
| Anti-mouse CD3 clone 17A2 BUV661 | BD | Cat# 741562; RRID: AB_2870988 |
| Anti-mouse CD8α clone 5H10-1 BUV805 | BD | Cat# 752640; RRID: AB_2917625 |
| Anti-mouse MHCII clone M5/114 BUV737 | BD | Cat# 569176; RRID: AB_3684842 |
| Anti-mouse CD69 clone H1.2F3 BUV563 | BD | Cat# 741234; RRID: AB_2870786 |
| Anti-mouse CD103 clone 2E7 BV785 | BioLegend | Cat# 121439; RRID: AB_2800588 |
| Anti-mouse CD25 clone PC61 PE/Dazzle 594 | BioLegend | Cat# 102048; RRID: AB_2564124 |
| Anti-mouse CD19 clone 6D5 BV605 | BioLegend | Cat# 115539; RRID: AB_11203538 |
| Anti-mouse F4/80 clone BM8 BV421 | BioLegend | Cat# 123137; RRID: AB_2563102 |
| Anti-mouse Ly6G clone 1A8 BV605 | BioLegend | Cat# 127639; RRID: AB_2565880 |
| Anti-mouse XCR1 clone ZET BV650 | BioLegend | Cat# 148220; RRID: AB_2566410 |
| Anti-mouse CD3 clone 17A2 BV605 | BioLegend | Cat# 100237; RRID: AB_2562039 |
| Anti-mouse CD86 clone GL1 BV421 | BioLegend | Cat# 105032; RRID: AB_2650895 |
| Anti-mouse/human CD11b clone M1/70 AF700 | BioLegend | Cat# 101222; RRID: AB_493705 |
| Anti-mouse/human CD11b clone M1/70 APC/FIRE750 | BioLegend | Cat# 101261; RRID: AB_2572121 |
| Anti-mouse CD8α clone 53-6.7 APC/FIRE750 | BioLegend | Cat# 100766; RRID: AB_2572113 |
| Anti-mouse/human Ki-67 clone SolA15 AF532 | Invitrogen | Cat# 58-5698-82; RRID: AB_ 2802365 |
| Anti-mouse CCR7 clone 4B12 APC/eFlour780 | Invitrogen | Cat# 47-1971-82; RRID: AB_2573974 |
| Anti-mouse CD11c clone N418 PE | Invitrogen | Cat# 12-0114-82; RRID: AB_465552 |
| Anti-mouse CD86 clone GL1 APC | Invitrogen | Cat# 17-0862-82; RRID: AB_469419 |
| Anti-mouse CCR7 clone 4B12 PE/Cy7 | Invitrogen | Cat# 25-1971-82; RRID: AB_469652 |
| Anti-mouse/human CD11b clone M1/70 APC/eFlour780 | Invitrogen | Cat# 47-0112-82; RRID: AB_1603193 |
| Anti-mouse CD3 clone 17A2 PerCP/eFlour710 | Invitrogen | Cat# 46-0032-82; RRID: AB_1834427 |
| Anti-mouse CD8α clone 53-6.7 APC/eFlour780 | Invitrogen | Cat# 47-0081-82; RRID: AB_1272185 |
| Anti-mouse CD45.2 clone 104 eFlour450 | Invitrogen | Cat# 48-0454-82; RRID: AB_11042125 |
| Fixable Live/Dead Blue | Invitrogen | Cat# L23105; UV Excitation |
| Fixable Live/Dead Aqua | Invitrogen | Cat# L34966; 405nm Excitation |
| Fc Block | Invitrogen | Cat# 14-0161-86; RRID: AB_467135 |
| Brilliant Stain Buffer | BD | Cat# 563794 |
| FACS Lysis | BD | Cat# 349202 |
